# Immune evasion mechanisms in early-stage I high-grade serous ovarian carcinoma: insights into regulatory T cell dynamics

**DOI:** 10.1038/s41419-025-07557-5

**Published:** 2025-04-01

**Authors:** Joanna Mikulak, Sara Terzoli, Paolo Marzano, Valentina Cazzetta, Giampaolo Martiniello, Rocco Piazza, Maria Estefania Viano, Domenico Vitobello, Rosalba Portuesi, Fabio Grizzi, Mohamed A. A. A. Hegazi, Barbara Fiamengo, Gianluca Basso, Lara Parachini, Laura Mannarino, Maurizio D’Incalci, Sergio Marchini, Domenico Mavilio

**Affiliations:** 1 Laboratory of Clinical and Experimental Immunology, IRCCS Humanitas Research Hospital, Rozzano, Milan, Italy; 2 Department of Biomedical Sciences, Humanitas University, Pieve Emanuele, Milan, Italy; 3 Department of Medical Biotechnology and Translational Medicine, University of Milan, Milan, Italy; 4 Department of Medicine and Surgery, University of Milan-Bicocca, Monza, Italy; 5 Unit of Gynecology, IRCCS Humanitas Research Hospital, Rozzano, Milan, Italy; 6 Department of Immunology and Inflammation, IRCCS Humanitas Research Hospital, Rozzano, Milan, Italy; 7 Unit of Pathological Anatomy, IRCCS Humanitas Research Hospital, Rozzano, Milan, Italy; 8 Humanitas Genomic Facility, IRCCS Humanitas Research Hospital, Rozzano, Milan, Italy; 9 Laboratory of Cancer Pharmacology, IRCCS Humanitas Research Hospital, Rozzano, Milano, Italy

**Keywords:** Ovarian cancer, Immune evasion, Immunosurveillance

## Abstract

The mechanisms driving immune evasion in early-stage I high-grade serous ovarian carcinoma (HGSOC) remain poorly understood. To investigate this, we performed single-cell RNA-sequencing analysis. Our findings revealed a highly immunosuppressive HGSOC microenvironment, characterized by abundant infiltration of regulatory T cells (Tregs). Trajectory analysis uncovered differentiation pathways of naïve Tregs, which underwent either activation and proliferation or transcriptional instability. The predicted Treg-cell interaction network, including crosstalk within tumor cells, facilitates Treg mobility and maturation while reinforcing their immunosuppressive function and persistence in the tumor. Moreover, their interactions with immune cells likely inhibit CD8 T cells and antigen-presenting cells, supporting tumor immune escape. Additionally, more immunogenic tumor conditions, marked by IFNγ production, may contribute to Treg destabilization. Our findings underscore the pivotal role of Tregs in early immune evasion of HGSOC and provide insights into potential therapeutic strategies targeting their activity and differentiation fate.

## Introduction

High-grade serous ovarian carcinoma (HGSOC) is the most prevalent and aggressive ovarian cancer (OC). A major challenge in managing HGSOC is its late-stage clinical detection, with ~80% of patients diagnosed at stage III-IV, which is associated with poor survival outcomes [1]. Identifying the key mechanisms underlying tumor evasion in the early stages of the disease could enable more effective, timely therapeutic interventions to prevent tumor progression and facilitate the identification of prognostic biomarkers.

The immune landscape in advanced-stage HGSOC encompasses both suppressive and protective immune mechanisms within the tumor microenvironment (TME). High levels of tumor-infiltrating lymphocytes (TILs) correlate positively with a favorable prognosis [2–6]. Conversely, the presence of tumor-associated macrophages (TAMs), and cancer-associated fibroblasts (CAFs) is linked to an immunosuppressive TME [7, 8]. Moreover, advanced-stage HGSOC is characterized by the infiltration of regulatory T cells (Tregs), with higher frequencies correlating with increased tumor burden and reduced survival outcomes [9–17]. In tumors, Tregs contribute to immune dysfunction and impaired tumor-associated antigen-specific immunity through various mechanisms, including the secretion of immunosuppressive cytokines (e.g., IL10, TGFB1), cytotoxic activity, induction of apoptosis via metabolic deprivation of IL2, and suppression of immune cell activation triggering immune-checkpoints such as CTLA4 [18, 19]. Consequently, several strategies targeting Tregs are being explored as promising approaches for cancer immunotherapy [20–22]. Recent advancements in transcriptomic single-cell RNA-sequencing (scRNA-seq) have provided deep insights into the mechanisms of immune evasion in advanced-stage HGSOC [23–25]. Despite this progress, the main molecular and cellular mechanisms governing immune escape at an early tumor stage remain poorly understood.

By applying scRNA-seq, we identified a significant infiltration, activation, and differentiation of Tregs in stage I HGSOC. This immunosuppressive state is associated with the dysfunction of cytotoxic CD8 T cells, natural killer cells (NKs), and dendritic cells (DCs), along with progressive tumor cell differentiation, which correlates with poor clinical outcomes. This study unveils key therapeutic targets to counteract immune escape mechanisms that emerge early in the disease.

## Materials and methods

### Patients’ enrolment and ethical statements

The collection of biological blood and tissue samples from patients diagnosed with HGSOC at stage I (FIGO), (Table 1), was ethically conducted following the Declaration of Helsinki and was approved by the Institutional Review Board (IRB) of HRH (Approval No. 225/20). All participants signed informed consent forms before participating in the study.

**Table 1:** HGSOC patient characterization.

Study participant	Age	FIGO stage	Histotype	Detected mutation	Clinical annotations	Conducted analysis
P1	63	IC	HGSOC	BRCA	Previous BC	scRNA-seq, IHC
P2	45	IA	HGSOC			scRNA-seq, IHC
P3	81	IC	HGSOC			IHC
P4	62	IC	HGSOC		Previous CRC	IHC
P5	54	IC	HGSOC			IHC
P6	58	IC	HGOSC			IHC
P7	53	IC	HGOSC			IHC
P8	51	IC	HGSOC			IHC
P9	53	IC	HGSOC			IHC
P10	57	IC	HGSOC			IHC
P11	57	IC	HGSOC	BRCA		IHC
P12	55	IC	HGSOC			IHC

*FIGO* The International Federation of Gynecology and Obstetrics, *BC* Breast cancer, *CRC* Colorectal cancer, *IHC* Immunochemistry, *scRNA-seq* single cell RNA-sequencing.

### Cell collection and processing

Freshly isolated ovarian tissues were processed for scRNA-seq experiments as follows: the tissues were dissociated using enzymatic and mechanical digestion in a gentleMACS Dissociator (Miltenyi, Germany) with RPMI-1640 supplemented with 1% FBS, 2 mg/mL collagenase D (Merck, Germany), and 100 μg/mL hyaluronidase (Merck). The resulting single-cell suspension was filtered through 100-μm and 70-μm cell strainers (Corning, USA), washed in HBSS^−/−^ (Euroclone SpA, Italy), and frozen in liquid nitrogen for further analysis. Upon thawing, the cells were stained for live/dead discrimination (Zombie Aqua^TM^ Fixable Viability Kit; BioLegend, USA), and with an anti-human CD45 monoclonal antibody (mAb) (Clone 5B1; Miltenyi; dilution 1:166). The CD45^+^ immune cells and tumor-associated CD45^−^ cell counterparts were then sorted using FACS Melody cell sorter (BD Biosciences). The sorted cells were immediately processed for scRNA-seq experiments.

PBMCs were isolated using Lympholyte®-H Cell Separation density gradient solution (Cedarlane Laboratories, USA) according to the manufacturer’s instructions and were frozen. Upon thawing, PBMC viability exceeded 95%, and the cells were immediately processed for scRNA-seq experiments.

### Library preparation and processing of scRNA-seq data

Libraries for scRNA-seq were prepared following the manufacturer’s guidelines using the Chromium Single Cell 3′ Library & Gel Bead Kit v3.1 (10x Genomics). For each sample, 10,000 target cells were loaded onto a Chromium single-cell controller (10X Genomics) to generate GEMs. Captured cells were lysed, and the released RNA was barcoded through reverse transcription in individual GEMs. Complementary (c)DNAs were amplified, and their quality was assessed using an Agilent 4200 Tapestation. The scRNA-seq libraries were sequenced with a paired-end 150-bp (PE150) reading strategy on the Illumina NextSeq500 sequencer for P1 and the Illumina NextSeq2000 sequencer for P2.

The scRNA-seq reads were aligned to the GRCh38 (version refdata-gex-GRCh38-2020-A, 10X Genomics) human genome reference, and UMIs were quantified using Cell Ranger 6.1.1 (10X Genomics). Subsequent analyzes were conducted using the Python package *Scanpy* (v1.9.3) under Python v3.9.7, unless not stated otherwise. Raw data matrices of all samples were merged, and cells with fewer than 400 expressed genes or 400 UMIs, or greater than 10% mitochondrial genes were removed. Data were log-normalized with a scale factor of 10,000, and highly variable genes (HVGs) were identified using Seurat dispersion-based methods. Principal component analysis (PCA) was performed using HVGs via ARPACK implementation of singular value decomposition (SVD).

To mitigate biases, multiple sample-data integration was achieved using the harmonypy package (v0.0.5), where the 6 samples were integrated using batch_id as the key covariate. Neighbors were identified using the top 6 components of Harmony-corrected PCA embeddings, and clustering was performed using the Leiden algorithm with an initial resolution of 2. Clusters were then embedded in two dimensions using Uniform Manifold Approximation and Projection (UMAP).

### Cell-cluster annotation

Differential expression gene (DEG) analysis between cell clusters was conducted to identify markers for each cell cluster using the *Wilcoxon rank-sum* test (*Scanpy*). Genes with an FDR-adjusted *P* value < 0.05 and expressed by at least 10% of cells in the cluster, with a minimum Log_2_-FoldChangeGene of |0.25|, were considered significant. A total of 21,697 cells were profiled and annotated based on the expression of canonical cell-type markers. Among them, 10,244 tumor-associated CD45^+^ immune cells, 5528 of their CD45^−^ tumor counterparts, and 5,925 of patient-matched PBMCs. Identified clusters of malignant cells, CD4^+^ T cells, cytotoxic lymphocytes, and myeloid cells were subjected to further independent scaling, dimensionality reduction, and unsupervised clustering, as described above.

### Pseudotime trajectory analysis

*Pseudotime* analysis was conducted by Monocle (v2.8.0) [26]. For malignant epithelial cells, individual trajectories were constructed for each patient using highly variable genes (HVGs) as ordering genes. Proliferative cells from each patient were designated as the root nodes of the graph. For tumor-infiltrating Tregs, a single trajectory was constructed for two patients using HVGs as the ordering genes, with the most naïve Tregs as the root node. The BEAM function was applied to identify genes with branch-dependent expression. Node 2 was selected as the branch point and a gene was defined as branch-dependent in its estimated q value was lower than 0.01. The expression of significantly branch-dependent genes was visualized on a heatmap using the *plot_genes_branched_heatmap()* function.

### Survival analysis

Survival analysis was conducted using *SurvivalGenie (*https://bbisr.shinyapps.winship.emory.edu/SurvivalGenie/) [27], a web tool designed to perform gene set survival analyzes across multiple cancer datasets available in The Cancer Genome Atlas (TCGA*;* http://cancergenome.nih.gov/) dataset. The TCGA was chosen for the advanced-stage OC dataset (*n* = 372). For malignant cells, survival analysis was performed on the gene set specific for each cell cluster obtained through DE analysis between all epithelial cell clusters separately for each patient. The top 35 DEGs for each cluster were selected based on adjusted *P* value < 0.01 and Log2 Fold-Change > 0.25 (Table S1). For tumor-infiltrating Tregs and cell-cell interaction analysis, survival analysis was conducted by uploading one specific gene or set genes. The *Cutp* method was utilized to define high and low groups. Results were visualized by Kaplan-Meier curves with corresponding Forest plots. *P* values were calculated using the log-rank test, with statistically significant differences denoted by *P* < 0.05.

### Single-cell gene regulatory network inference

Single-cell gene regulatory network (GNR) inference was performed on tumor-infiltrating Tregs by using *pySCENIC* package (v0.11.2) [28], a Python implementation of the *SCENIC* pipeline. GNR was inferred using the GRNBoost2 algorithm, given a predefined list of TFs (All_TFs_hg38.txt). Candidate regulons (i.e., a TF and its predicted target genes) from TF-target gene interaction, motif enrichment, and TF-regulon prediction were computed by using the *pyscenic cxt* function. The activity of the predicted regulons (consisting of the TF and their putative target genes) in the individual cells was computed by using the *AUCell* method. The default values were used for all parameters. The cluster-specific regulons were identified by the *regulon_specificity_scores()* function. The regulon specificity score (RSS) (from 0 to 1, 1 indicating complete specificity of the regulon for the cell type), was calculated for each cell type separately, and the top 10 regulons were shown for each cluster.

### RNA-velocity analysis

*RNA-velocity* analysis [29] was performed using the Python package *scVelo* (v0.2.4) to infer the future states of a cell from unspliced and spliced mRNA counts. The matrices of spliced and unspliced counts were quantified using the Python library *velocyto* (v0.17) from the aligned bam file generated by Cell Ranger. Counts matrices in loom file format were loaded to scVelo and merged into the anndata object. Genes detected in less than 20 unspliced and spliced counts were filtered out, and the counts were then normalized. The *filter_genes_dispersion()* function was used to identify the top 4000 HVGs. *The scv.pp.moments()* function was used to compute the first- and second-order moments among nearest neighbors in the PCA space. The velocities and velocity graphs were estimated using the generalized dynamical model (*scv.tl.recover_dynamics(), scv.tl.velocity()*, *scv.tl.velocity_graph()*). Velocities were visualized on UMAP embedding.

### Functional enrichment analysis

Reactome enrichment analysis was performed using the *Reactome* webtool (https://reactome.org/), while KEGG pathway functional enrichment was performed by the R package *clusterProfiler* (v4.8.3) [30]. The pathways of cell types were enriched using DEGs with FDR-adjusted *P* value < 0.05 and Log_2_-FoldChange > |0.25|. Only enriched terms with FDR (Reactome) or *q*-value (KEGG) < 0.05 were selected as significant.

### Cell-cell communication analysis

Cell-cell interaction analysis was performed using *nichenetr* (v2.0.1), the R implementation of the NicheNet method [31]. The NicheNet analysis was applied to investigate the cell-cell interactions between different cell type subpopulations and specific Treg cell subsets, acting as either the sender or receiver populations. Only genes expressed in at least 10% of the sender/receiver population were considered. Marker genes for each receiver population were identified based on adjusted *P* values < 0.05 and Log2-FoldChange > 0.5. Ligand activities were predicted using the predict_ligand_activities() function and ranked by the Pearson correlation coefficient. The ligand-receptor network was then predicted based on prior interaction potential, and the top 25 ligand-receptor pairs were visualized by dot plots.

### Proliferation scores

Specific proliferation scores, including S and G2M scores, were computed by summing the scaled expression values of a list of cell cycle marker genes **(**Table S1**)** using the Scanpy-implemented *scanpy.tl.score_genes()* function. To evaluate the cell cycle phase, initially, a score for each cell was calculated based on the expression of G2M and S phase markers using the *scanpy.tl.score_genes_cell_cycle()* function. Cells expressing neither of these markers were associated with the G1 phase [32].

### Lipid-associated, pro-angiogenesis, and inflammatory scores

The specific myeloid scores, which include the lipid-associated (LA), pro-angiogenesis (Angio), and inflammatory (Inflam) scores were computed, for each myeloid cell group by summing the scaled expression values of LA marker gene list (*n* = *19* genes), pro-Angio. marker gene list (*n* = *20* genes), and Inflam. marker gene list (*n* = *21* genes) respectively (Table S2) [33]. Scores were calculated using the Scanpy implemented *scanpy.tl.score_genes()* function and visualized by radar plots.

### Immunohistochemistry staining and analysis

Hematoxylin and eosin (H&E) and immunohistochemistry (IHC) staining studies were performed on 3 μm-thick formalin-fixed paraffin-embedded tumor sections of 11 HGSOC patients. IHC experiments were performed on 3 μm-thick formalin-fixed paraffin-embedded sections (FFPE) of HGSOC tissues. Slides underwent deparaffinization, rehydration, and heat-induced antigen retrieval using Diva Decloaker solution (Biocare Medical, USA) in a Decloaking Chamber (Biocare Medical) at 125 °C for 3 min and at 90 °C for 5 min according to manufacturer’s instructions. After rinsing in H_2_O, endogenous peroxidase inhibition for FOXP3 staining was achieved using 3% H_2_O_2_ (Merck) for 10 min at RT following antigen retrieval. For CD4 staining, this inhibition step was applied after primary Ab incubation. Sections were then blocked with Background Sniper (Biocare Medical) for 20 min at RT and incubated with anti-human CD4 mAb (clone UMAB64; Origene, USA; dilution 1:150) to detect membrane surface expression of the CD4 protein. Alternatively, sections were incubated with anti-human FOXP3 mAb (Clone 86D; Biocare Medical; dilution 1:100) to stain intranuclear presence of the FOXP3 protein. Tissue sections were further incubated with MACH1 Universal HRP-Polymer Detection (Biocare Medical) following manufacturer’s instructions. The chromogen reaction was developed using 3,3′-Diaminobenzidine tetrahydrochloride (DAB; Biocare Medical), and the sections were counterstained with hematoxylin (Dako Agilent Technologies, USA). Immunostained sections were captured by digitized at 20x objective magnification using a Zeiss Axioscan.Z1 (Zeiss, Germany).

For quantitative analysis, detection of CD4 and FOXP3 positive areas was measured in three randomly selected and non-contiguous microscopic tumor regions of interest (ROI) for each patient (P1-P11). The histopathologists, blinded to any patient clinical data, selected the areas of interest. Digital images (15729×15201 pixels; 0.22 pixel/micron resolution), covering ~35 mm^2^ each, were captured. Automatic detection of CD4 and FOXP3 markers was performed based on the software Cell Cycle Spatial Analyzer, Version 1.0. To measure the extent of CD4 and FOXP3 immunoreactivity, we used a computer-aided image-analysis system capable of distinguishing the immunostained areas based on red, green, and blue color segmentation, and calculating the relative percentage of immunoreactive area to the total digitally captured tissue area. Due to the challenges posed by dense infiltrates or agglomerates, for accurate optical counting of CD4^+^ cells, converting area values to cell counts was not attempted. The mean of values obtained from three ROIs was calculated for each tumor and utilized for subsequent data analysis. The distribution of CD4^+^ and FOXP3^+^ cells in a two-dimensional pattern was automatically evaluated using a “quadrat count” algorithm, which determines the level of cells spatial heterogeneity [34]. The relative dispersion (RD) indicative of the percentage of the inter-ROI and Intra-ROI variability was calculated by the formula:

$${RD}=\left(\frac{{SD}}{{mean}}\right)x100$$

### Statistical analyses

Statistical analyses of scRNA-seq data were performed using GraphPad PRISM software version 10.2.0 (La Jolla, California, USA). *P* values < 0.05 were considered statistically significant. Specific statistical methods used are detailed in the figure legends.

## Results

### Dominant infiltration of Treg cells in stage I HGSOC

**Fig. 1:**
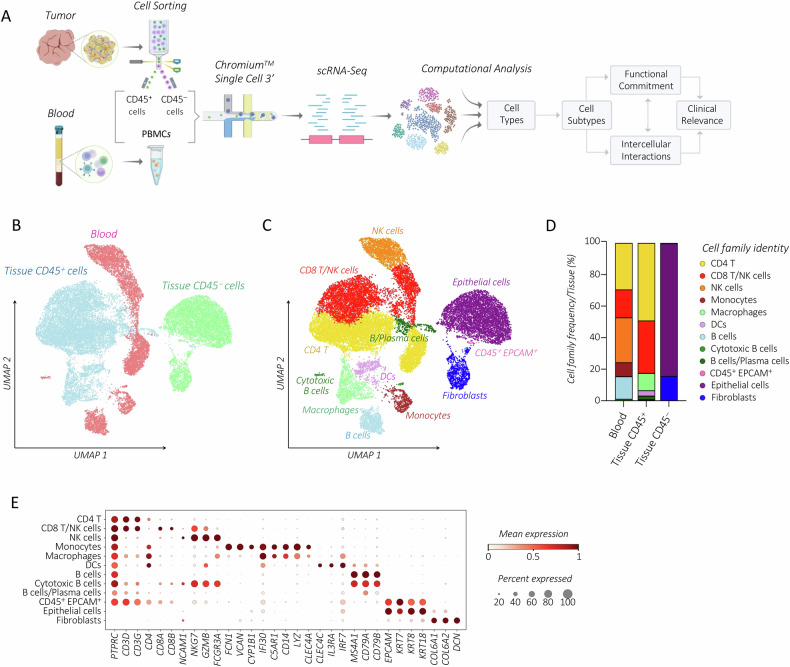
Integrated scRNA-seq analysis reveals heterogeneous immune cell composition in the stage I HGSOC. **A** Workflow illustrating the processing of blood and tumor samples followed by experimental and analytical procedures. *UMAP* visualization of all 21,697 integrated cells derived from tumor-associated CD45^+^ immune cells, their CD45^−^ tumor counterparts, and matched PBMCs. Cells are color-coded based on tissue origin (**B**) or cell types **(C**). **D** Bar plot showing the frequency (%) of each cell type detected among blood and tumor-derived CD45^+^ and CD45^−^ cells in two patients. Cell numbers were normalized to the total loaded cell number for each tissue-origin. **E** Dot plot displaying the expression of canonical gene markers used for annotation of blood and tumor-associated CD45^+^ and CD45^−^ cell types. Dots are colored by the average expression of each gene scaled across all clusters and sized by the percentage of cells within a cluster (min.pct ≥ 10%).

We conducted scRNA-seq analysis on tumor lesions and PBMCs from two incidentally diagnosed stage I HGSOC patients who underwent ovarian resection without prior chemotherapy (Table 1 and Fig. S1A). To perform detailed immune cell characterization, scRNA-seq analysis was conducted on FACS-sorted tumor-associated CD45^+^ immune cells and their CD45^−^ tumor counterparts (Figs. 1A, and S1B). Transcriptional expression profiles were merged across all tissues and patients, retaining 21,697 cells (Fig. 1B). Of these cells, 25.5%, 47.2%, and 27.3% originated from CD45^−^ tumor-tissue cells, intratumoral CD45^+^ immune cells, and blood samples, respectively. Major cell types included immune cells, fibroblasts, and epithelial cells (Fig. 1C–E). Among immune cells, we detected a dominant population of CD4 T cells, along with CD8 T cells, NKs, B/Plasma cells, monocytes/macrophages, and DCs (Figs. 1D, and S1C).

**Fig. 2:**
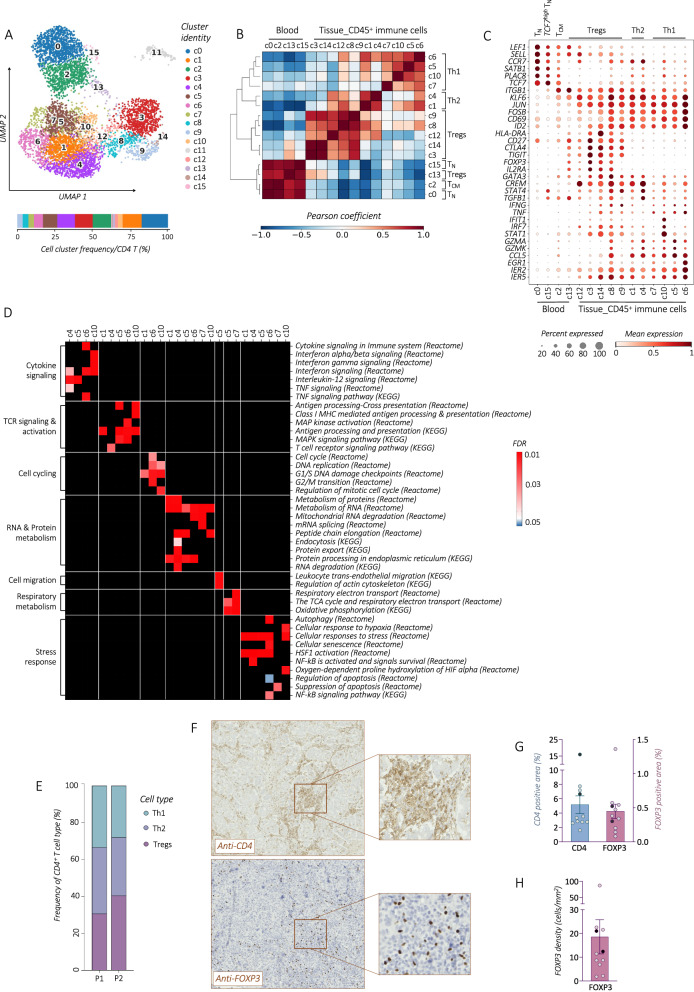
Dissecting the complexity of tumor-infiltrating CD4 T lymphocytes in stage I HGSOC. **A** *UMAP* visualization (upper panel) with relative frequency (%) distribution (lower panel) of re-clustered total blood and tumor-infiltrating CD4 T cell subsets. **B**, **C** Profiling of blood and tumor-infiltrating CD4 T cell subsets. Heatmap showing the Pearson correlation matrix for different CD4 T cell subsets (**B**), dot plot showing the expression of key selected differentiation and activating gene markers (**C**), dots are colored by the average expression of each gene scaled across all clusters and sized by the percentage of cells within a cluster (min.pct ≥ 10%)**. D** Heatmap displaying the selection of significantly enriched *Reactome* and *KEGG* pathways with *FDR*-value < 0.05 (*Reactome*) or *q*-value (*KEGG*) < 0.05, identified among DEGs (refer to the Method section) in different CD4 T cell subtypes. **E** Bar plot showing the relative frequencies (%) of Th1, Th2, and Treg subtypes among tumor-infiltrating CD4 T lymphocytes analyzed for each patient, P1 and P2. Cell numbers were normalized to the total number of tumor-infiltrating CD4 T lymphocyte for each patient. **F** One representative IHC staining of CD4 and FOXP3 proteins in stage-I HGSOC (*out of 11*). IHC study showing statistical analysis of the mean ( ± SEM) of immunopositive CD4^+^ and FOXP3^+^ areas (**G**), and total FOXP3^+^ cell density (**H**), represented as dot plots (*n* = *11*). Dots highlighted in dark blue (CD4) and dark purple (FOXP3) indicate samples analyzed by scRNA-seq.

To dissect the diversity of CD4 T cells, further analysis was performed resulting in 15 cell clusters (c0-c10, c12-c15) (Figs. 2A, and S2A). Cells in c11 were excluded from further CD4 T cell analysis due to the lack of expression of CD3 and CD4, as well as transcriptional profile resembling Plasma cells (i.e., *CD79A*, *SDC1, JCHAIN)* (Fig. S2B). A hierarchical clustering analysis reflected high CD4 dissimilarities between blood and tumor cells (Fig. 2B). In the blood, CD4 T cells were predominantly composed of naïve (T_N_) cells in c0, of *TCF7*^*high*^ T_N_ in c15, and central memory (T_CM_) cells in c2. Blood c13 comprised resting Tregs expressing canonical markers *FOXP3, CTLA4, TIGIT*, and *IL2RA*, along with the T_N_ profile (*SELL, LEF1*).

In the tumors, CD4 TILs showed patterns of Th1, Th2 and Treg subtypes, all displaying an activating profile (*ITGB1*, *KLF6*, *JUN*, *FOSB*, *CD69*, *ID2*, and *HLA-DRA*) (Fig. 2C). In particular, Th1 cells in c6 presented an early activated pattern (*EGR1*, *IER2/5*). Cells in c10 manifested the IFN-induced profile (*IFIT1*, *IRF7*, *STAT1)*. The increased cytotoxic profile detected in c4-c5 reflects highly differentiated T_EMRA_ cells (*GZMK*, *GZMA*, *CCL5*). According to these observations, biological pathway analysis (*Reactome, KEGG*) showed different effector Th1 and Th2 cell states indicative of immunogenic tumor, resulting in a TCR activation, antigen presentation, proliferation, and interferon-induced signaling. Moreover, we detected status of trans-endothelial migration, as well as activation of protein and respiratory metabolisms and stress-induced hypoxia-responsive patterns (Fig. 2D).

Within the tumor, Tregs were identified across multiple cell subsets (c3, c8-c9, c12, and c14) based on the expression of canonical markers and differentiation correlation analysis (Fig. 2B, C). Indeed, among total CD4 TILs, the frequency of Tregs reached a mean of 35% and was consistent between the two patients (Fig. 2E). To further confirm Treg infiltration in stage I tumors, we conducted quantitative tissue analysis on formalin-fixed paraffin-embedded (FFPE) tumor blocks of 11 stage I HGSOC patients (Table 1). We applied standardized immunohistochemistry (IHC) technique combined with a computer-aided image analysis system to explore the expression of CD4 and FOXP3 proteins (Fig. 2F). Analyzed tumor regions (ROIs), exhibited 29.5% ( ± 10.1 SEM) of carcinogenic heterogeneity with a similar inter-ROI and intra-ROI mean heterogeneity for CD4 and FOXP3 protein distribution (Fig. S2C, D). The immunoreactive FOXP3^+^ area covered ~10% of the total CD4^+^ zones (0.45% ± 1.1 SEM vs. 5.2% ± 1.24 SEM) (Fig. 2G) and corresponded to 18.6 cells/mm2 (±7.2 SEM) FOXP3^+^ cells mean density (Fig. 2H). No significant correlation was observed between CD4^+^ and FOXP3^+^ immunoreactive areas, indicating the independent tumor infiltration of these two cell types (Fig. S2E).

### Dynamic remodeling of Treg cells in stage I HGSOC

**Fig. 3:**
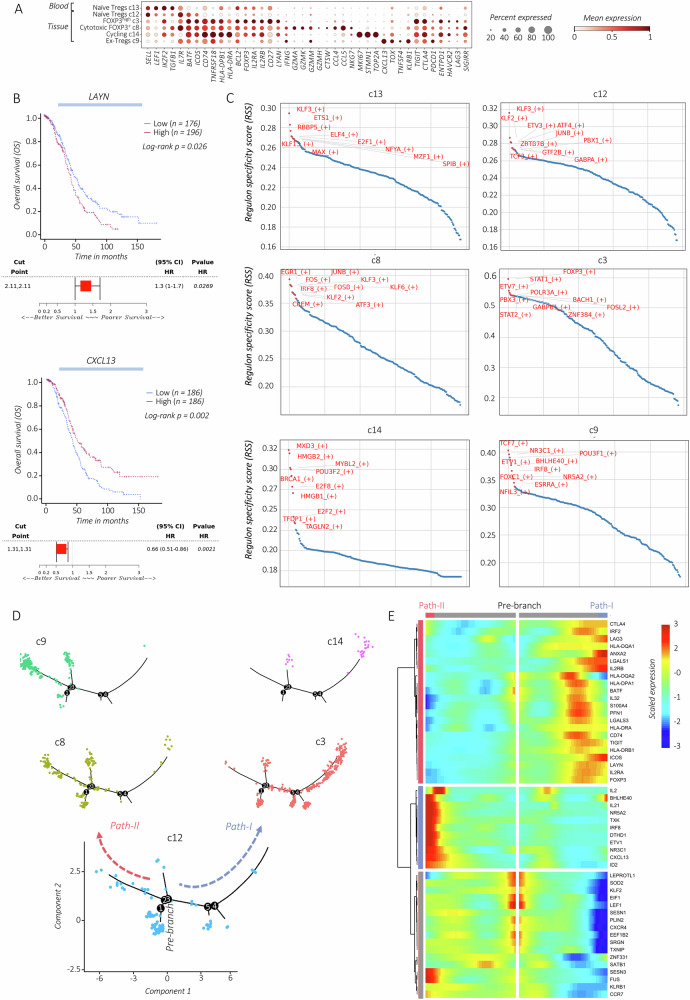
Dissecting the heterogeneity of Treg cells in stage I HGSOC. **A** Dot plot displaying the expression of selected gene markers used for different Treg subsets annotation. Dots are colored by the average expression of each gene scaled across all clusters and sized by the percentage of cells within a cluster (min.pct ≥ 10%). **B** Kaplan–Meier curves with corresponding Forest plots for patients with advanced OC (TCGA dataset), demonstrating OS differences between high-risk and low-risk expression of *LAYN* (upper panel) and *CXCL13* (lower panel). Significant differences between the two groups are indicated by the *P* value < 0.05. **C** The top 10 predicted transcription factors (TFs) driving the activation of different tumor-infiltrating Treg subsets in c3/8/9/12/13/14. The TFs are ranked by their specificity score shown on the *y*-axis, ranging from 0 to 1, with 1 indicating complete specificity. **D** Pseudotime trajectory of distinct tumor-infiltrating Treg subtypes for each patient (P1, P2), colored by clusters c3/8/9/12/14. The arrows represent the two trajectory paths starting from naïve Tregs in c12: Path-I leading to *FOXP3*^*high*^ Tregs in c3 and proliferative Tregs in c14, and Path-II leading to *FOXP3*^*+*^ Tregs in c8 and to ex-Tregs in c9. **E** Heatmap of the top 50 significantly branch-dependent genes (*q*-value < 0.01) variable along the two pseudotime Path-I and Path-II. The *x*-axis represents cells ordered by pseudotime values along Path-I (from middle to right), and along Path-II (from middle to left), and different colors correspond to the scaled (Z-scored) expression of each gene in each cell.

Tregs in stage I HGSOC show distinct transcriptomic profiles (Figs. 3A, and S3A). Cells in c12 exhibit a *SELL*^*high*^*FOXP3*^*low*^*CD25*^*low*^*CTLA4*^*low*^ phenotype, indicating c12 as the most naïve Treg status in the tumor. Expression of transcription factor (TF) *IKZF2* (Helios), a marker of thymic-derived Tregs, was detected in all *FOXP3*^*+*^ Tregs, indicating their thymic origin. Cells in c3 show a hyperactive *FOXP3*^*high*^*CD25*^*high*^*CTLA4*^*high*^ immunosuppressive profile, similar to that described in multiple other solid tumors [35, 36]. Tregs in c3 expressed high levels of *LAYN* (Layilin), a protein linked to the motility and suppressive function of Tregs in tumors [35], and related to poor prognosis in stage-advanced HGSOC patients analyzed using the Cancer Genome Atlas (TCGA) datasets (Fig. 3B). Moreover, cells in c3 show synergistically higher levels of immune-checkpoints *HAVCR2* (TIM3), *LAG3* and *ENTPD1* (CD39), whose expression in Tregs, similar to CTLA4, is associated with increased immune-suppressive activity [37] (Fig. 3A). Conversely, cells in c3 show low expression of *PDCD1* (PD-1), which, similarly to its role in conventional T cells, inhibits Treg activity [38, 39]. The *IKZF2*^*+*^*FOXP3*^*+*^*CD25*^*+*^*CTLA4*^*high*^ Tregs in c14 present proliferative *MKI67*^*high*^ signature (Figs. 2A, and S3B; Table S1). The *IKZF2*^*+*^*FOXP3*^*+*^*CD25*^*+*^*CTLA4*^*+*^ Treg profile in c8 was associated with increased cytotoxicity (i.e., *GZMA, GZMM, GZMK, GZMH, CTSW, NKG7)*, and the potential secretion of the immunosuppressive cytokine *TGFB1* and the lymphocyte recruiting *CCL4/5* chemokines secretion.

Cells in c9 show a *KLRB1*^*high*^*FOXP3*^*−*^*CD25*^*−*^*CTLA4*^*+*^ profile, presenting features of Treg instability (ex-Tregs). The transcriptional affinity of ex-Tregs with other Treg subsets in our dataset was indicated by hierarchical analysis (Fig. 2B), and the expression of several Treg-associated genes such as *BATF, TIGIT, CTLA4, ICOS, BCL2, TNFRSF18 (GITR), IL2RB, CD27, CD74*, and *HLA-DPB1* (Fig. 3A). Transcriptional re-programming can lead to loss of Treg fate marked by loss of *FOXP3* and *IKZF2* expression, and functional reprogramming [21, 40]. However, the factors driving ex-Treg differentiation remain poorly understood. These cells express elevated levels of *PD-1*, which, in the tumor setting, has been linked to the loss of Treg cell suppressive functions, and similar to our observation, increased secretion of IFNγ [41]. Furthermore, cells in c9 display high levels of *CXCL13*, a chemokine strongly associated with better OS in stage III/IV OC patients, based on TCGA dataset analysis (Fig. 3B). This suggests that Tregs in c9 not only lose their *FOXP3*^*+*^ immunosuppressive profile but also acquire anti-tumoral effector functions. Notably, the prognostic benefits of CXCL13 expression in HGSOC were linked to the PD-1 blockade therapy and organization of the tumor-associated tertiary lymphoid structures [42, 43].

To further analyze the diversity in the transcriptional regulation of Tregs, we used the *SCENIC* tool. The top 10 active TFs with activating and repressor gene programs detected for each Treg subset are shown in (Fig. 3C). Expression of transcriptional repressors including *ETS1* and the members of Krüppel-like (KLF) family (e.g., *KLF2, KLF3*) in c13 and c12 Tregs show naïve phenotype of Tregs. Contrarily, the expression of *FOXP3* and the *STAT*-gene family in c3 confirmed Treg genetic stability [18, 44]. Likewise, the enriched expression of the *AP-1* TF family, exhibiting transcriptional activity in the *FOXP3* promoter, indicates the transcriptional stability of Tregs in c8. In addition, c8 expresses *EGR1* which in turn promotes *TGFB1* expression [45], and *IRF8* involved in Treg-mediated control of the Th1 response [46]. The TF pattern in c9 supports their de-differentiation by the expression of *TCF1* (TCF7), a repressor of *FOXP3*, and *BHLHE40*, a positive regulator of *CXCL13* expression in Tregs [44, 47]. Moreover, their expression of *IRF8* links their transcriptional re-programming to anti-tumor Th1 activity.

To analyze the differentiation status across tumor-associated Tregs (Fig. 3D), placing the most naïve Tregs at the beginning of the *Pseudotime* trajectory, we observed that the initial state in c12 bifurcates into two main branches dissected by the specific gene changes along the pre-branch naïve Tregs, Path-I and Path-II (Fig. 3D, E). Path-I consists of hyperactive *FOXP3*^*high*^*CD25*^*high*^*CTLA4*^*high*^ cells in c3, terminating with *FOXP3*^*+*^*MIK67*^*high*^ Tregs. On the other hand, Path-II consists of cytotoxic *FOXP3*^*+*^*CD25*^*+*^*CTLA4*^*+*^ cells in c8 and the *CXCL13*^*high*^*KRB1*^*high*^*FOXP3*^*−*^ ex-Tregs.

Anti-CD25, anti-CCR4, and anti-CCR8 Abs are currently being considered for Treg depletion in various tumors [48]. We then analyzed the expression of different cytokine receptors in our dataset. In more differentiated Treg subsets, we found high expression of *CXCR3, CCR4*, and *IL2RB* (Fig. S3C). Moreover, mature *FOXP3*^*+*^ Tregs in the tumor express *CXCR6, IL21R, IL1R1, IL18R1*, and, to a lesser extent, *CCR8*.

### Cytotoxic lymphocyte profiling in stage I HGSOC

**Fig. 4:**
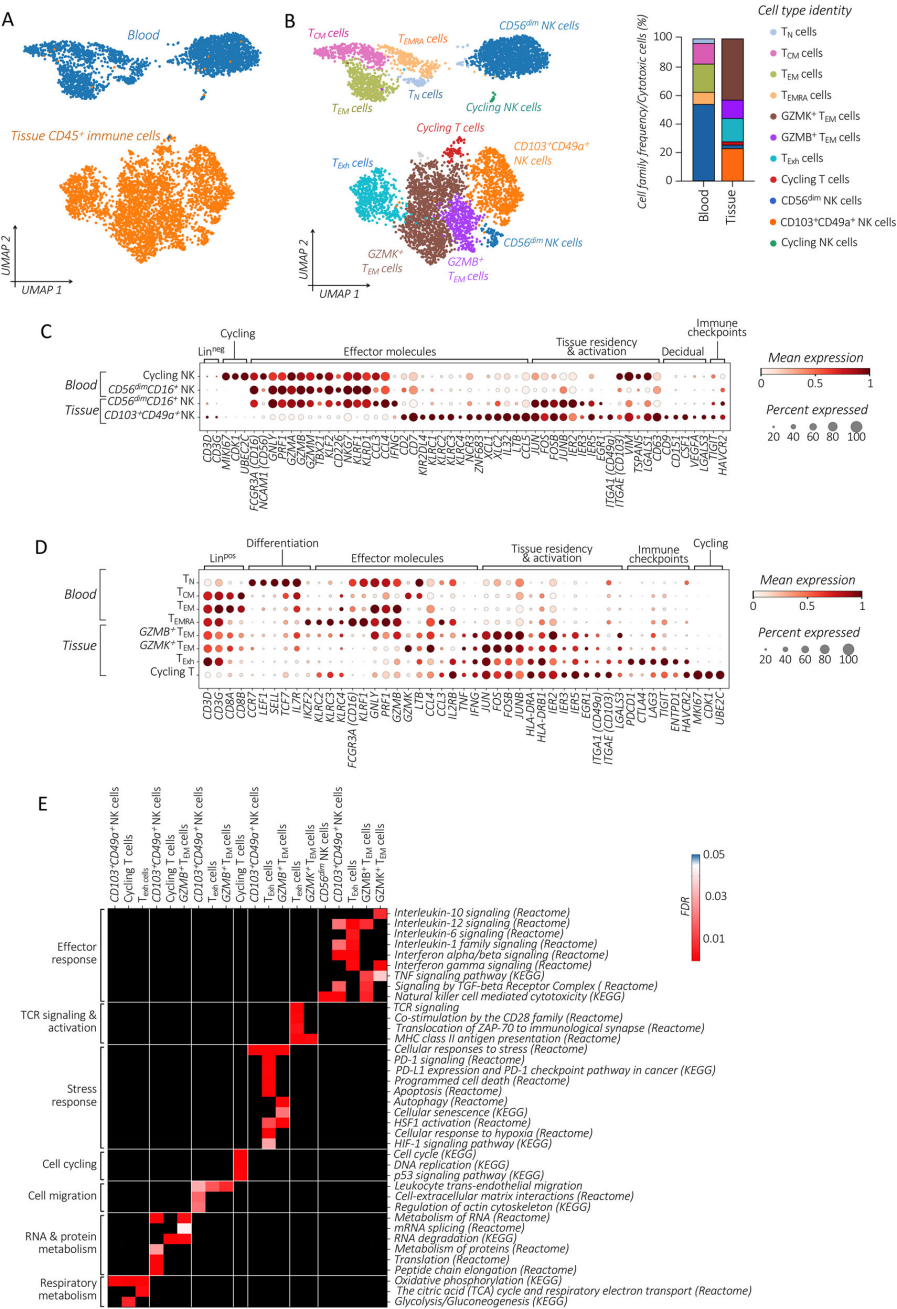
Profiling of cytotoxic lymphocytes in stage I HGSOC. *UMAP* visualization of re-clustered blood and tumor-associated cytotoxic CD8 T and NK cells, colored by tissue origin (**A**), or by cell types (**B**; left panel), alongside the corresponding relative frequency (%) distribution (right panel) of each cell subtype for tissue-origin. Profiling of blood and tumor-infiltrating CD8 T (**C**) and NK cell subsets (**D**) by analyzing the expression of key selected differentiation and effector genes depicting various cell patterns: cycling, exhausted T (T_Exh_), T_N_, T_EM_, T_CM_, T_EMRA_, NK CD56^dim^ (CD56^dim^CD16^+^), NK decidual-like (*CD9*^*+*^*CD151*^*+*^*CSF1*^*+*^*VEGFA*^*+*^*LGALS3*^*+*^) and NK tissue-resident (*CD49a*^*+*^*CD103*^*+*^); displayed in dot plots. Dots are colored by the average expression of each gene scaled across all clusters and sized by the percentage of cells within a cluster (min.pct ≥ 10%). **E** Heatmap displaying the selection of significantly enriched *Reactome* and *KEGG* pathways with *FDR*-value < 0.05 (*Reactome*) or *q*-value (*KEGG*) < 0.05, identified among DEGs (refer to the Method section) in different CD8 T and NK cell subtypes.

Tumor-infiltrating NKs exhibit a distinctive profile compared to their blood-circulating counterparts (Figs. 4A–C, and S4). We identified two NK cell subsets within the tumor. Among them, the smaller *CD56*^*dim*^*CD16*^*+*^ NK cell subset, which, similar to the blood, exhibits high cytotoxicity (*PRF1, GZMB/A/M, GNLY, TBX21*), and cytokine secretion (*IFNG, CCL3/4*) potential. Moreover, their profile is marked by the expression of several activating receptors *KLRD1* (CD94), *KLRF1, CD226* (DNAM1), and *NKG7*. On the other hand, the predominant tumor-associated NKs display low cytolytic potential, along with the expression of the inhibitory receptor *KLRC1* (NKG2A). Similar to findings in advanced-stage HGSOC, they express tissue-resident markers CD49a (*ITGA1*) and CD103 (*ITGAE*), and resemble a pro-angiogenic decidual (d)NK-like profile *(CSF1, VEGFA, CD9, CD151*) [49–51]. Notably, we observed increased expression of tissue-residence markers (*ITGA1, VIM, LGALS1*) also in proliferating blood NKs, indicating their inclination toward blood-tumor migration.

Analyzing CD8 TILs, we focused on the expression of *ENTPD1* (CD39), an indicator of the tumor-associated response [52]. Importantly, *CD39*^*+*^ CD8 T cells presented a highly exhausted profile, as evidenced by the expression of *PD-1(PDCD1), CTLA4, LAG3, TIGIT*, and *HAVCR2* (TIM3) (Fig. 4B, D). Although to a lesser extent, *CD39* expression was also detected on cycling *MKI67*^*high*^ CD8 TILs, providing evidence of tumor-specific activation. On the other hand, the lack of *CD39* expression was observed in two CD8 T_EM_ subsets, *GZMK*^*high*^ and *GZMB*^*high*^, among which *GZMK*^*high*^ CD8 T_EM_ phenotype constitutes the most abundant subset with low lytic potential (*PRF1, GLNY*, *GZMB*.) Notably, multiple lines of evidence have linked the CD8 T_EM_ *GZMK*^*high*^ phenotype to tumor progression [53]. The pathway enrichment analysis highlighted TCR-signaling activation and proliferation, dynamic metabolic changes, as well as the status of CD8 T exhaustion (Fig. 4E).

### Myeloid cell analysis in stage I HGSOC

**Fig. 5:**
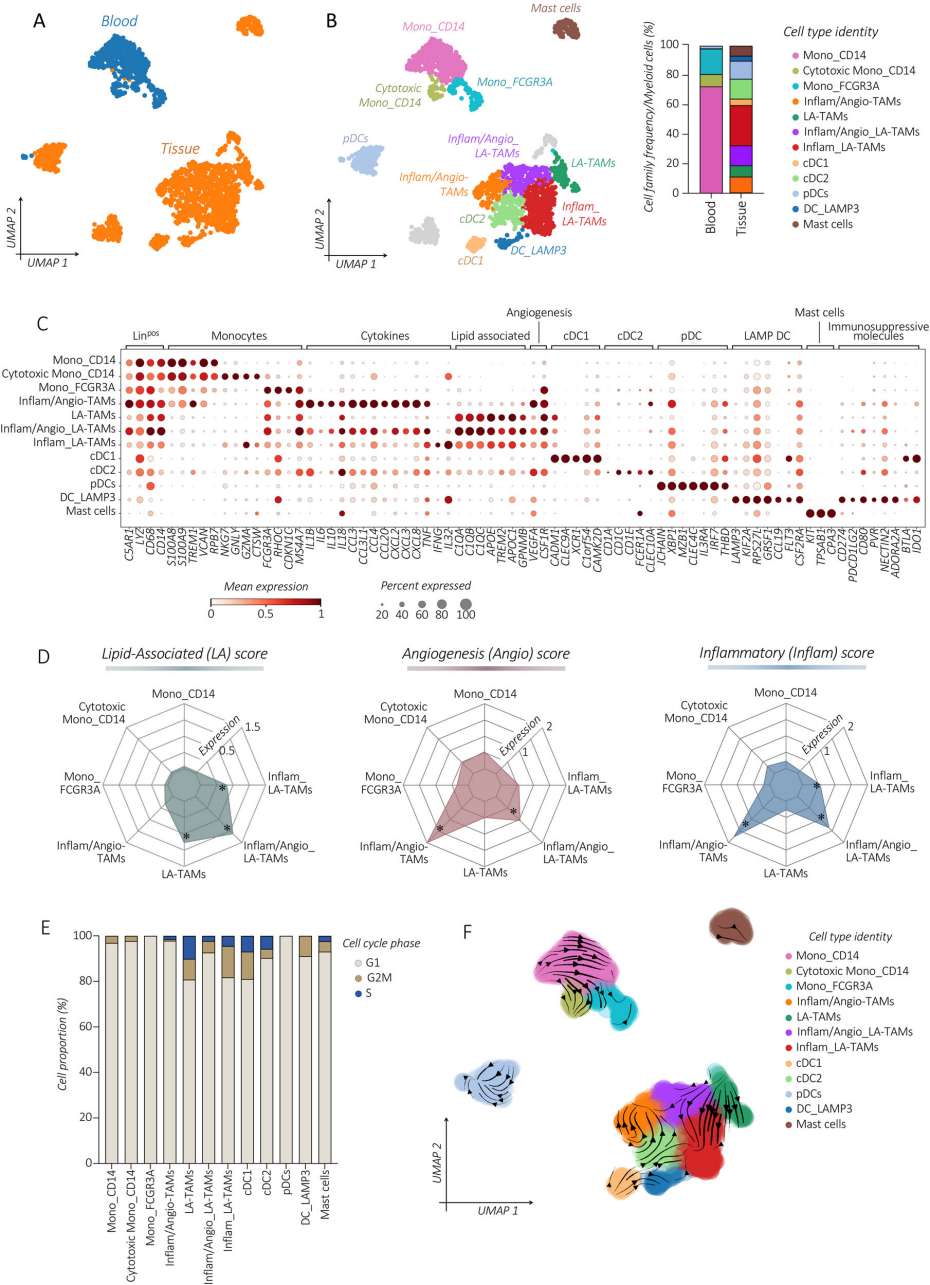
Profiling of myeloid cell subsets in stage I HGSOC. *UMAP* visualization of re-clustered blood monocytes (Mono) and tumor-associated macrophages (TAMs), dendritic cells (DCs), and Mast cells, colored by tissue origin (**A**) or by cell type subsets (**B**; left panel), alongside the corresponding relative frequency (%) distribution (right panel) of each cell type for tissue-origin. **C** Dot plot showing profiling of blood and tumor-associated myeloid cells analyzed by the expression of key selected differentiation and effector gene markers depicting specific cell patterns: *CD14*^*+*^, *CD16*^*+*^ (FCGR3A), inflammatory (Inflam), pro-angiogenesis (Angio), lipid-associated (LA), *IFNG*^*+*^ conventional (c)DCs, plasmacytoid (p)DCs and *LAMP3*^*+*^. Dots are colored by the average expression of each gene scaled across all clusters and sized by the percentage of cells within a cluster (min.pct ≥ 10%). **D** Radial graphs showing the expression level of the specific lipid-associated, pro-angiogenesis, and inflammatory gene module scores (refer to the Method section) calculated for each myeloid cell group. **E** Bar plot illustrating the relative frequencies (%) of specific cell cycle phase G1, G2M, and S distribution in all detected myeloid cell subsets. **F***UMAP* showing the inferred development dynamics of myeloid cell subsets by *RNA-velocity*.

The blood comprises subsets of classical *CD14*^*+*^*CD16*^*−*^, cytotoxic *CD14*^*+*^*CD16*^*low*^, and non-classical *CD14*^*low*^*CD16*^*high*^ monocytes (Figs. 5A–C and S5). Given the complexity of TAMs within HGSOC lesions, we utilized specific gene-score signatures to delineate their functional diversity. This includes lipid-associated (*n* = *19* genes), pro-angiogenesis (*n* = *20* genes), and inflammatory (*n* = *21* genes) gene module scores (Table S2) [33]. The prevailing profile detected in TAMs exhibited a lipid-associated (LA)-pattern (e.g., *APOE, TREM2, APOC1, C1QA, C1QB, GPNMB*) (Fig. 5C, D). Combining *RNA-velocity* analysis with the proliferation profile, we observed that the LA-TAM subset, boasting a relatively higher proliferation score, undergoes further polarization, giving rise to the inflammatory (Inflam)_LA-TAM (e.g., *TNF, IL1B, IL18, IL32, CCL3/4*), and to a pro-angiogenic (Angio)/Inflam_LA-TAM (e.g., *VEGFA, CXCL8, CSFR1*) subsets (Fig. 5E, F and Table S1). Thus, we support the notion that lipid-associated metabolism dysregulation is a hallmark of TAMs in stage I HGSOC.

Among conventional (c)DCs, we distinguished cDC1, cDC2, and plasmacytoid (p)DCs (Fig. 5C). We also identified mature *LAMP3*^*+*^ DCs, expressing markers *CD80* and *CD83*, along with *CCR7*, pointing their ability to migrate towards lymph nodes and activate immune cells (*CCL16/19/22, IL15*) [54]. Mature *LAMP3*^*+*^ DCs may originate from cDC1 and cDC2. In our dataset, maturation dynamics indicate a predominant maturation of the cDC1 subset, which accordingly constitutes the most proliferative DC subset (Fig. 5E, F).

### Characterization of malignant cells

**Fig. 6:**
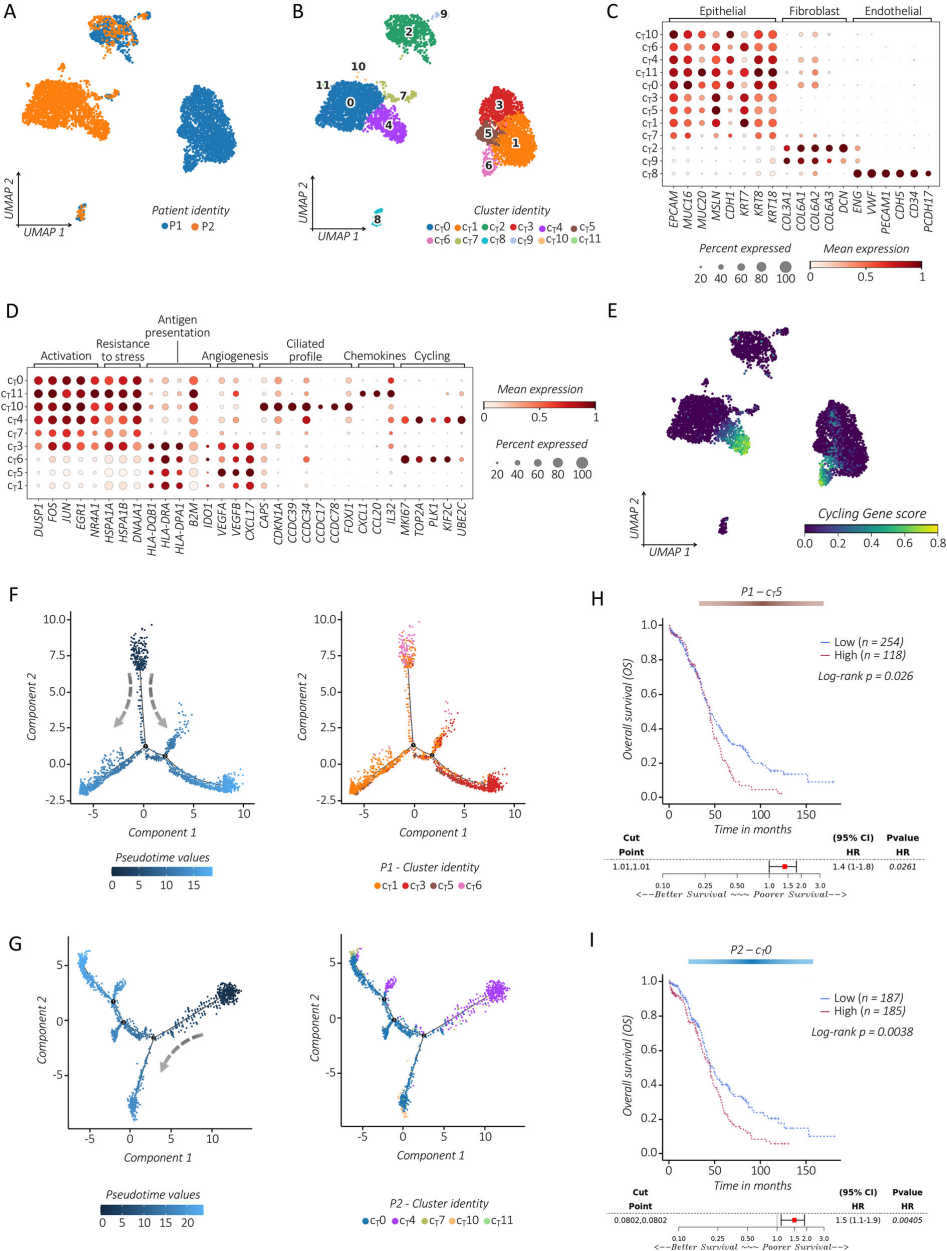
Dissecting the heterogeneity of tumor cells in stage I HGSOC. *UMAP* visualization of tumor-derived CD45^−^ cells analyzed in the two stage I HGSOC lesions. Cells are colored by patient (**A**) or cluster identity (c_T_0-c_T_11) (**B**). **C** Dot plot displaying the expression of canonical markers used for annotating different tumor-associated cell types. **D** Dot plot showing the expression of selected gene markers for each malignant epithelial cell cluster. Dots are colored by the average expression of each gene scaled across all clusters and sized by the percentage of cells within a cluster (min.pct ≥ 10%). **E** Feature plot depicting the expression of cycling-gene score (refer to the Method section). Pseudotime trajectory of malignant epithelial cells for P1 (**F**) and P2 (**G**). Each cell is colored by its pseudotime values from dark to light blue (right panels) or by cluster identity (left panels). The arrows indicate the initial point of the trajectory starting from proliferative cells in c6 and c4 for P1 and P2, respectively. Kaplan–Meier curves with corresponding Forest plots for patients with advanced OC (TCGA dataset), showing OS differences between high- and low-risk patients separated based on the specific 35 top DEGs obtained separately for c_T_5 (**H**) and c_T_0 (**I**). Significant differences between groups are indicated by the *P* value < 0.05.

CD45^−^ cells were re-clustered and divided into 12 distinct cell clusters (c_T_0-c_T_11) (Figs. 6A–C and S6**)**. We found the transcriptional similarities between fibroblast (c_T_2 and c_T_9), and endothelial cells (c_T_8) in two patients (Figs. 6A–C and S7A). Conversely, malignant epithelial cells exhibited patient-specific profiles. Fibroblasts showed low expression of CAF markers (*FAP, MMP11*, *WNT5, IL24, IL6)* (Fig. S7B). Malignant epithelial cells expressed secretory markers including *EPCAM*, cytokeratins (*KRT8/10/18*), *MUC16*, the carrier of the CA125 tumor marker, and mesothelin (*MSLN*), an OC differentiation antigen (Fig. 6C). When evaluating proliferation rates of tumor cells, c_T_4, and c_T_6 emerged as the most actively cycling cells in P1 and P2, respectively (Fig. 6D, E). The activation status (*DUSPs, AP-1s, EGRs, NR4A1/2/3/4*), stress resistance (e.g., *HSPA1A/1B, DNAJA1*), and antigen presentation (*HLA-DQB1, HLA-DRA*) varied among patients (Fig. 6D). Pro-angiogenic properties were marked in c_T_5 (*VEGFA/B* and *CXCL17*). Notably, features of ciliated cells, reminiscent of the fallopian epithelium, were evident in c_T_10 and c_T_6, which expressed coiled-coil domains containing (*CCDC*) proteins [55]. The expression of epithelial-mesenchymal transformation (EMT)-associated genes (e.g., *MYH14, CDH1, CDH6, HOXD1, SOX5/11/17*) were detected in cT0 and cT4. However, the canonical EMT markers (e.g., *TWIST1/2, SNAI1/2*) were absent (Fig. S7C). Functional enrichment pathway analysis (*Reactome, KEGG*) confirmed multiple pathway activations, including cytokine signaling, antigen presentation, proliferation, pro-angiogenic activity, and mitochondrial alterations (Fig. S7D). Moreover, a shared cilium-epithelium-related pathway was observed in c_T_10, as well as in the proliferating subsets c_T_4 and c_T_6.

By exploring the pseudotime trajectory analysis, and patient-specific tumor cell transcriptomic profiles in correlation with OS prognosis in the ATCC dataset, employing the *Survival Genie* web tool, we analyzed malignant cell progression (Figs. 6F–I, and S7E, F). For each analysis, we plotted a Kaplan–Meier curve based on the specific tumor cell cluster-associated signature, using the top 35 up-regulated DEGs (Table S3). Our findings revealed that patient-specific tumor cells in c_T_0 and c_T_5 were significantly associated with a worse prognosis (Fig. 6G–I).

Therefore, we concluded that stage I tumors in the two examined patients share cycling epithelial-ciliated profiles with progressive malignant transformation, despite immune pressure, likely due to immune escape.

### Mapping Treg cell interactions in stage I HGSOC

**Fig. 7:**
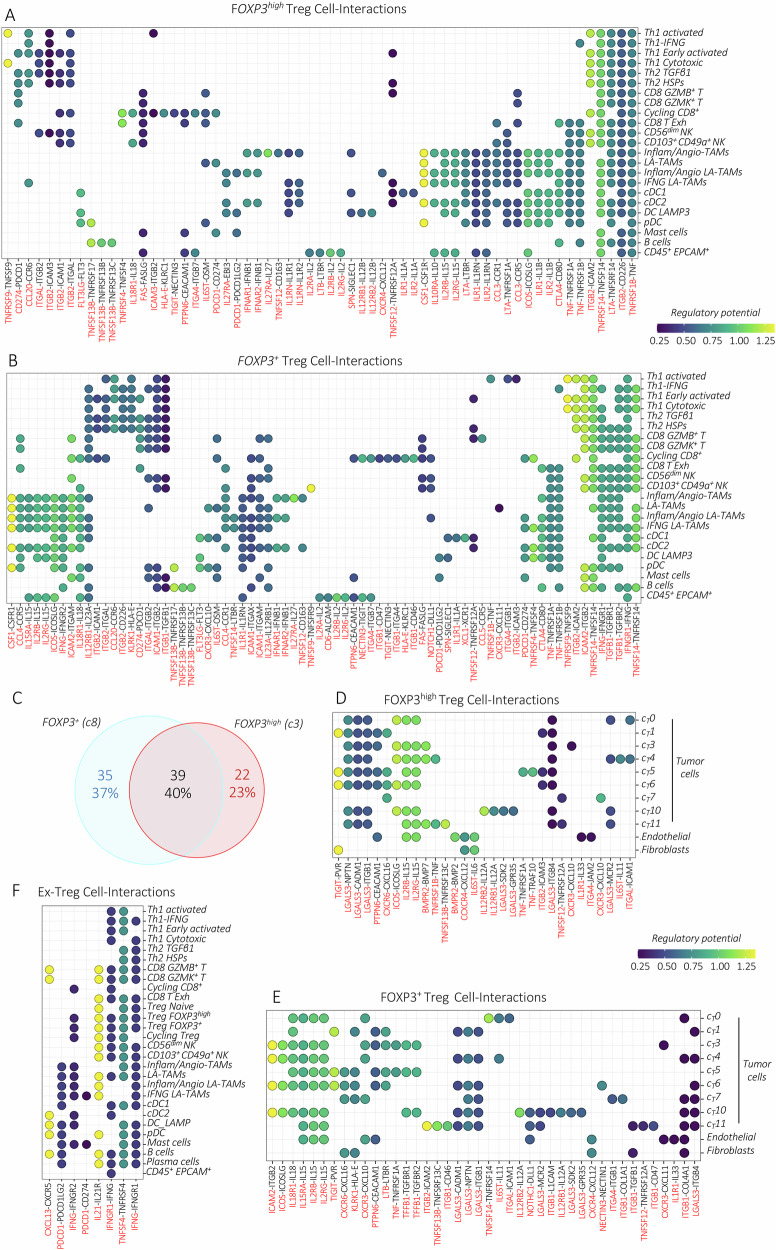
Treg cells shape the cellular interaction in stage I HGSOC. Dot plots displaying ligand-receptor interactions on the *y*-axis, showcasing the top 25 prioritized (by Pearson correlation coefficient) paired ligand-receptor interactions of *FOXP3*^*high*^ Tregs (**A**) and *FOXP3*^*+*^ Tregs (**B**) with immune cell family. Dots are colored according to the regulatory potential values. Ligands or receptors expressed in Tregs (in red) are matched with their corresponding receptors or ligands (in black) expressed on different cell subsets shown on the *x*-axis. **C** The Venn diagram shows the overlapping or specifically paired ligand-receptor engagements among different cell types and *FOXP3*^*high*^ and *FOXP3*^*+*^ Tregs. Dot plots displaying ligand-receptor interactions on the *y*-axis, showcasing the top 25 prioritized (by Pearson correlation coefficient) paired ligand-receptor interactions of *FOXP3*^*high*^ Tregs (**D**) and *FOXP3*^*+*^ Tregs with cancer cells (**E**). Dots are colored according to the regulatory potential values. Ligands or receptors expressed in Tregs (in red) are matched with their corresponding receptors or ligands (in black) expressed on different cell subsets shown on the *x*-axis. (**F**) Dot plot showing selected paired ligand-receptor engagements detected in ex-Tregs.

To predict Treg communication network we employed the *NicheNet* algorithm. Focusing our analysis on the top 25 ranked Treg ligand-receptor pairs, we identified multiple connections with T and myeloid cell subsets shared between c3 and c8 Treg subsets (Fig. 7A–C). Importantly, the interaction of CD80 with CTLA4, expressed on Tregs, was detected in DCs, B, and CD8 T cells, predicting their inhibition. Moreover, the interaction of TGFβ1 released by *FOXP3*^*+*^ Tregs can broadly inhibit DCs, CD8 T, and NK cells targeting the TGFBR1/2 receptors [18, 37]. The IL2RA/RB-IL2 connection was enriched between *FOXP3*^*high/+*^ Tregs and the *CD45*^*+*^*EPCAM*^*+*^ cells, providing evidence of the IL2 Treg-mediated deprivation within the TME. Interactions between *FOXP3*^*high/+*^ Tregs and various immune cell subsets including ligand-receptor pairs: ICOS-ICOSLG, IL18R1-IL18, ILR1-IL1B, Notch1-DLL1, IL15R/IL2RA-IL15, IL27RA-IL27/EBI3, IL1R1-IL33, IL12RB1-IL23A, and TNFRSF1B-TNF and TNFRSF9-TNFSF9, also may play important roles in Treg cell immunosuppressive activity, increased transcriptomic stability, and promoting their maturation and expansion [18, 56–61]. Moreover, Treg interactions through the CXCR3, CXCR4, ICAMs, and integrins, in particular ITGB2, mobilize and activate Tregs [18, 62, 63]. Several of the ligand-receptor connections listed above were detected between *FOXP3*^*high/+*^ Tregs and cancer cells, as well (e.g., ICOS-ICOSLG, IL12RB1/B2-IL12, ILR1-IL33, CXCR3-CXCL10/CXCL11, CXCR4-CXCL12) (Fig. 7D, E). Additionally, interactions between *FOXP3*^*high/+*^ Tregs and cancer cells may be involved in Treg mobilization (CXCR6/CXCL16) and activity (TIGIT/PVR) [64, 65]. Reciprocally, *LGALS3*, a marker of human Tregs, may interact with multiple ligands detected on tumor cells, supporting HGSOC progression [66]. Analyzing the impact of the ligand-receptor gene axes, detected in our *FOXP3*-expressing Tregs, on clinical outcomes in TCGA-OC dataset we found several interactions that correlate with worse OS (Fig. S8).

The most prevalent interaction of ex-Tregs involves the reciprocal IFNG-IFNGR interactions (Fig. 7F). Ex-Tregs bearing *IFNGRs* exhibit interactions with other abundantly *IFNG*-expressing cell types, including *FOXP3-*expressing Tregs, NK and T cells. In this context, recent observations indicate IFNγ as a driver of Treg fragility [41]. Of note, higher expression of *IFNG* in TCGA-OC patients is associated with better OS (Fig. S8). Similarly, the specific secretion of IL21 by ex-Tregs that can react with different immune cells targeting IL21R is associated with better survival rates in OC patients analyzed from the TCGA dataset (Fig. S8). Conversely, *PD-1* expression in ex-Tregs predicted enriched interactions with its ligands PD-L1/L2 (*CD274/PDCD1LG2*) within myeloid and *FOXP3*-expressing Treg subsets, possibly suggesting their inhibition.

## Discussion

We present a comprehensive atlas of the single-cell transcriptome of stage I HGSOC, providing detailed insights into the immune cells, and highlighting their molecular signatures and intercellular communication patterns. We detected high Treg heterogeneity in stage I HGSOC. Despite differences in patient-specific tumor cell characteristics, ICH analysis confirmed consistent Treg infiltration in most stage I HGSOC, supporting the notion of a common feature of early tumors favoring Treg accumulation. A majority of human solid tumors exhibit a high presence of FOXP3^high^ Tregs [48]. Similarly, highly suppressive *FOXP3*^*high*^ Tregs have been identified in advanced-staged OC and linked to a worse prognosis [19, 67]. Our study showed evidence of their role in supporting HGSOC during early growth. Moreover, we identified that the *FOXP3*^*high*^ subset predominantly expresses *LAYN*, a marker that defines highly TCR-activated Tregs and is associated with poor prognosis in OC [68]. *FOXP3*^*+*^ Treg suppressive activity may be conferred by a combination of cytotoxic activity, secretion of suppressive TGFB1, and, similar to the *FOXP3*^*high*^ subset, by the suppressive activity exhibited by IL2RA and CTLA4 receptors. We found that all *FOXP3*-expressing Tregs show a thymic origin by expressing *IKZF2* (Helios), a TF that stabilizes the inhibitory activity of Treg. In addition, we identified the ex-Tregs, likely arising from transcriptional program instability due to the loss of *FOXP3* expression. Certain pro-inflammatory signals, such as IFNγ, can cause a rapid loss of suppressor activity, and re-programming of the Tregs into a pro-inflammatory phenotype [21, 41, 69–72]. Interestingly, exploring the TFs associated with Treg differentiation, we found that Path-II displays a high expression of IFN-regulatory factor 8 (*IRF8*), a downstream target of IFNγ-signaling in Th1-controlled response [73, 74]. Intriguingly, *IRF8* expression was commonly observed among the top TFs in *FOXP3*^*+*^ and ex-Treg subsets, suggesting its involvement in promoting Path-II Treg profiles. Moreover, the expression of both *IFNG* and its receptors *IFNGR1/2*, reported to be involved in Treg destabilization [41], was detected across cell-cell interactions specifically within the *FOXP3*^*+*^ and ex-Treg subsets. These data suggest IFNγ as a possible driver for Treg instability in stage I HGSOC, as observed in melanoma and head and neck squamous cell carcinoma [41]. These data suggest that more immunogenic malignant cell states resulting in a higher IFNγ-mediated immune response could be predictive of Treg dysregulation. In support of this hypothesis, we observed that the instability of Tregs leads to the expression of CXCL13, a chemokine responsible for the recruitment of B cells and T cells to the tumor site [75]. In advanced-stage HGSOC, CXCL13 expression correlates with prolonged patient survival and the initial formation of tertiary lymphoid structures (TLS) [42], coordinating the antitumor response. Our study, reveals that the dominant producer of CXCL13 are ex-Tregs, thereby supporting the notion that Treg instability may contribute to immune-boosting in HGSOC. Importantly, increased CXCL13 expression was found in neoantigen-reactive T cells, an observation that extends across various tumor types [76]. Therefore, future research exploring the relationship between tumor cell immunogenicity, specific T cell tumor reactivity, and Treg stability could provide a deeper understanding of immune response regulation in the HGSOC.

In stage I TME, we observed the infiltration of other immune cells, including TAMs, and DCs, as well as canonical cytotoxic populations of CD8 T and NK cells. As mentioned earlier, the activation of CD8 T cells leads to heightened exhaustion. Notably, NK cells exhibit primarily a tissue-resident *CD103*^*+*^*CD49*^*+*^*KLRC1*^*+*^ and dNK-like profile with low cytotoxic capabilities. The similar *CD103*^*+*^*CD49*^*+*^*KLRC1*^*+*^ profile, however, with the absence of cytotoxic potential, distinguishes these NK cells from those found in ascites of advanced-stage metastatic HGSOC [50]. Intriguingly, NKG2A (*KLRC1*) receptor was proposed to “educate” uterine NK cells by enhancing their effector responses [77]. Thus, delving into the role of KLRC1 aligned with the potential acquisition of cytotoxicity by NK cells during tumor progression would be intriguing.

Several lines of evidence suggest that immunotherapy in the early stages of cancer is linked to a higher frequency of expanded T cell clones, greater immune diversity, and reduced immunosuppression, supporting the hypothesis that early intervention may enhance therapeutic efficacy [78–80]. Our study reveals that in stage I HGSOC, the TME is shaped by continuous interactions between tumor cells and immune effectors, creating a delicate balance between activation and suppression. A distinct subset of tumor-reactive, proliferative CD8 T cells, and cytotoxic NK cells emerge, yet the tumor persists, protected by immune escape mechanisms. Tregs play a central role in this immunosuppressive network. To target this network, we identified CCR8, IL2R, and CCR4, markers currently under clinical evaluation for Treg depletion [48], along with CXCR6, IL18R1, and IL21R, potential novel targets for disrupting Treg-mediated suppression. Additionally, our findings highlight IFNγ signaling as a key modulator of Treg function, opening new avenues for their functional reprogramming [21, 22]. While early diagnosis of HGSOC remains a challenge, by redefining its landscape, our study provides valuable insights that inform the development of novel biomarkers and targeted therapeutic strategies.

We acknowledge several limitations of our study, including the small number of scRNA-seq samples, which stems from the rarity of early-stage I HGSOC and its asymptomatic nature. However, the statistical robustness of scRNA-seq analysis allows for meaningful insights even with limited sample sizes. Moreover, to address this limitation, we performed a targeted ICH analysis, confirming the presence of Tregs in stage I HGSOC. Additionally, we supplemented our analysis with TCGA data from advanced stages, revealing similarities in specific gene expression patterns, suggesting that certain features of advanced tumors may already be present in early-stage disease. Another limitation relates to the predicted interactions generated by *NicheNet*. While *NicheNet* is a powerful tool for identifying multiple cell-cell interactions and generating experimental hypotheses, further targeted experimentation is required to validate these predictions and ensure the scientific integrity of our work

In summary, our study provides profound insights into the immunosuppressive TME of early-stage I HGSOC. Specifically, we identified notable Treg heterogeneity and uncovered previously unrecognized dual pathways of Treg polarization in HGSOC, providing important insights for future investigations and potential therapeutic interventions.

## Supplementary information

Supplementary Material

## Data Availability

All data needed to evaluate the conclusions in the paper are present in the paper and/or the Supplementary Materials. The scRNA-seq data are available from the Gene Expression Omnibus (GEO) repository (https://www.ncbi.nlm.nih.gov/geo/), accession code GSE290141. Data from TCGA dataset used in this work can be accessed at “http://cancergenome.nih.gov/”. R and Python scripts enabling the main steps of the analysis are available upon request to the corresponding authors.
