# Correction: Immune evasion mechanisms in early-stage I high-grade serous ovarian carcinoma: insights into regulatory T cell dynamics

**DOI:** 10.1038/s41419-025-07701-1

**Published:** 2025-05-16

**Authors:** Joanna Mikulak, Sara Terzoli, Paolo Marzano, Valentina Cazzetta, Giampaolo Martiniello, Rocco Piazza, Maria Estefania Viano, Domenico Vitobello, Rosalba Portuesi, Fabio Grizzi, Mohamed A. A. A. Hegazi, Barbara Fiamengo, Gianluca Basso, Lara Parachini, Laura Mannarino, Maurizio D’Incalci, Sergio Marchini, Domenico Mavilio

**Affiliations:** 1 Laboratory of Clinical and Experimental Immunology, IRCCS Humanitas Research Hospital, Rozzano, Milan, Italy; 2 Department of Biomedical Sciences, Humanitas University, Pieve Emanuele, Milan, Italy; 3 Department of Medical Biotechnology and Translational Medicine, University of Milan, Milan, Italy; 4 Department of Medicine and Surgery, University of Milan-Bicocca, Monza, Italy; 5 Unit of Gynecology, IRCCS Humanitas Research Hospital, Rozzano, Milan, Italy; 6 Department of Immunology and Inflammation, IRCCS Humanitas Research Hospital, Rozzano, Milan, Italy; 7 Unit of Pathological Anatomy, IRCCS Humanitas Research Hospital, Rozzano, Milan, Italy; 8 Humanitas Genomic Facility, IRCCS Humanitas Research Hospital, Rozzano, Milan, Italy; 9 Laboratory of Cancer Pharmacology, IRCCS Humanitas Research Hospital, Rozzano, Milano, Italy

**Keywords:** Ovarian cancer, Immune evasion, Immunosurveillance

Correction to: *Cell Death and Disease* 10.1038/s41419-025-07557-5; published online 1 April 2025

In this article the Y-axis titles in fig. 2G and 2H are not visible. This has been corrected.
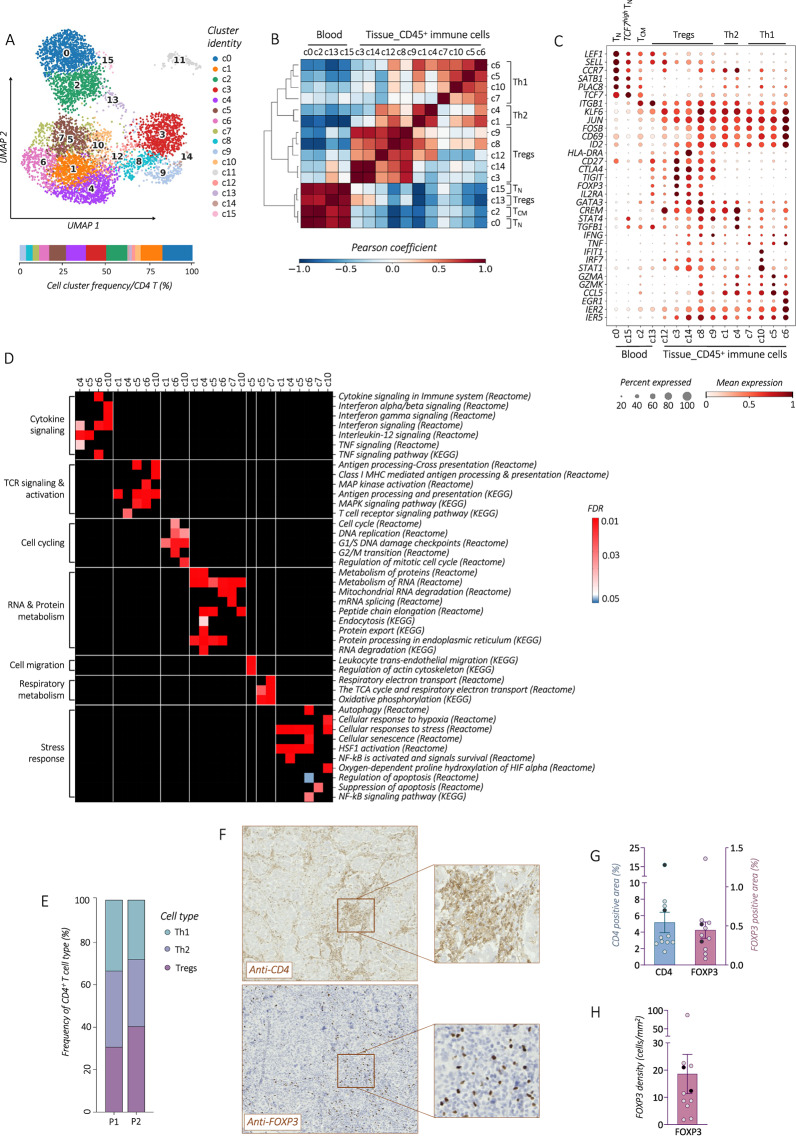


The original article has been corrected.

