# Supplementary material for: Immune evasion mechanisms in early-stage I high-grade serous ovarian carcinoma: insights into regulatory T cell dynamics
Source: Cell Death Dis. 2025 Apr 1;16(1):229. doi: 10.1038/s41419-025-07557-5 (PMC11958665; doi:10.1038/s41419-025-07557-5)
Supplement: Supplementary file 1 — Supplementary Material [file 41419_2025_7557_MOESM1_ESM.pdf]

Supplementary Material

Immune Evasion Mechanisms in Early Stage-I High-Grade Serous Ovarian Carcinoma: Insights into Regulatory T-Cell Dynamics

Joanna Mikulak [1], Sara Terzoli [1,2], Paolo Marzano [1,3], Valentina Cazzetta [1,3], Giampaolo Martiniello[3], Rocco Piazza [4], Maria Estefania Viano [3], Domenico Vitobello [5], Rosalba Portuesi [5], Fabio Grizzi [6], Mohamed A.A.A. Hegazi [6], Barbara Fiamengo [7], Gianluca Basso [8], Lara Parachini [2,9], Laura Mannarino [2,9], Maurizio D'Incalci [2,9], Sergio Marchini [9]† and Domenico Mavilio [1,3] †**

*†Shared last-authorship; *Corresponding author*

[1]Laboratory of Clinical and Experimental Immunology, IRCCS Humanitas Research Hospital, Rozzano, Milan, Italy;
[2]Department of Biomedical Sciences, Humanitas University, Pieve Emanuele, Milan, Italy;
[3]Department of Medical Biotechnology and Translational Medicine, University of Milan, Milan, Italy;
[4]Department of Medicine and Surgery, University of Milan-Bicocca, Monza, Italy;
[5]Unit of Gynecology, IRCCS Humanitas Research Hospital, Rozzano, Milan, Italy.
[6]Department of Immunology and Inflammation, IRCCS Humanitas Research Hospital, Rozzano, Milan, Italy;
[7]Unit of Pathological Anatomy, IRCCS Humanitas Research Hospital, Rozzano, Milan, Italy;
[8]Humanitas Genomic Facility, IRCCS Humanitas Research Hospital, Rozzano, Milan, Italy.
[9]Laboratory of Cancer Pharmacology, IRCCS Humanitas Research Hospital, Rozzano, Milano, Italy.

List of Supplementary Figures and Tables

1) **Supplementary Figures:**

Figure S1. Comparative analysis of the stage I HGSOC microenvironment in two patients.

Figure S2. Detection of tumor-infiltrating CD4 T lymphocytes in stage I HGSOC.

Figure S3. Transcriptomic diversity of Treg cell subsets in stage I HGSOC.

Figure S4. Detection of cytotoxic lymphocytes in stage I HGSOC

Figure S5. Detection of myeloid cells in stage I HGSOC

Figure S6. Transcriptomic patient-dependent diversity of tumor cells in stage I HGSOC.

Figure S7. Transcriptomic profiling of tumor cell subtypes in stage I HGSOC

Figure S8. Clinical relevance of the predicted Treg cell-interaction network in stage I HGSOC

2) **Supplementary Tables:**

Table S1. Cell cycle phase gene list

Table S2. Gene-score signatures used to compute the Lipid-Associated (LA), Angiogenesis (Angio) and Inflammatory (Inflam) effector scores in myeloid cells

Table S3. Specific tumor cell cluster-associated signature in stage I HGSOC patients P1 and P2

Supplementary Figures and Tables with Legends

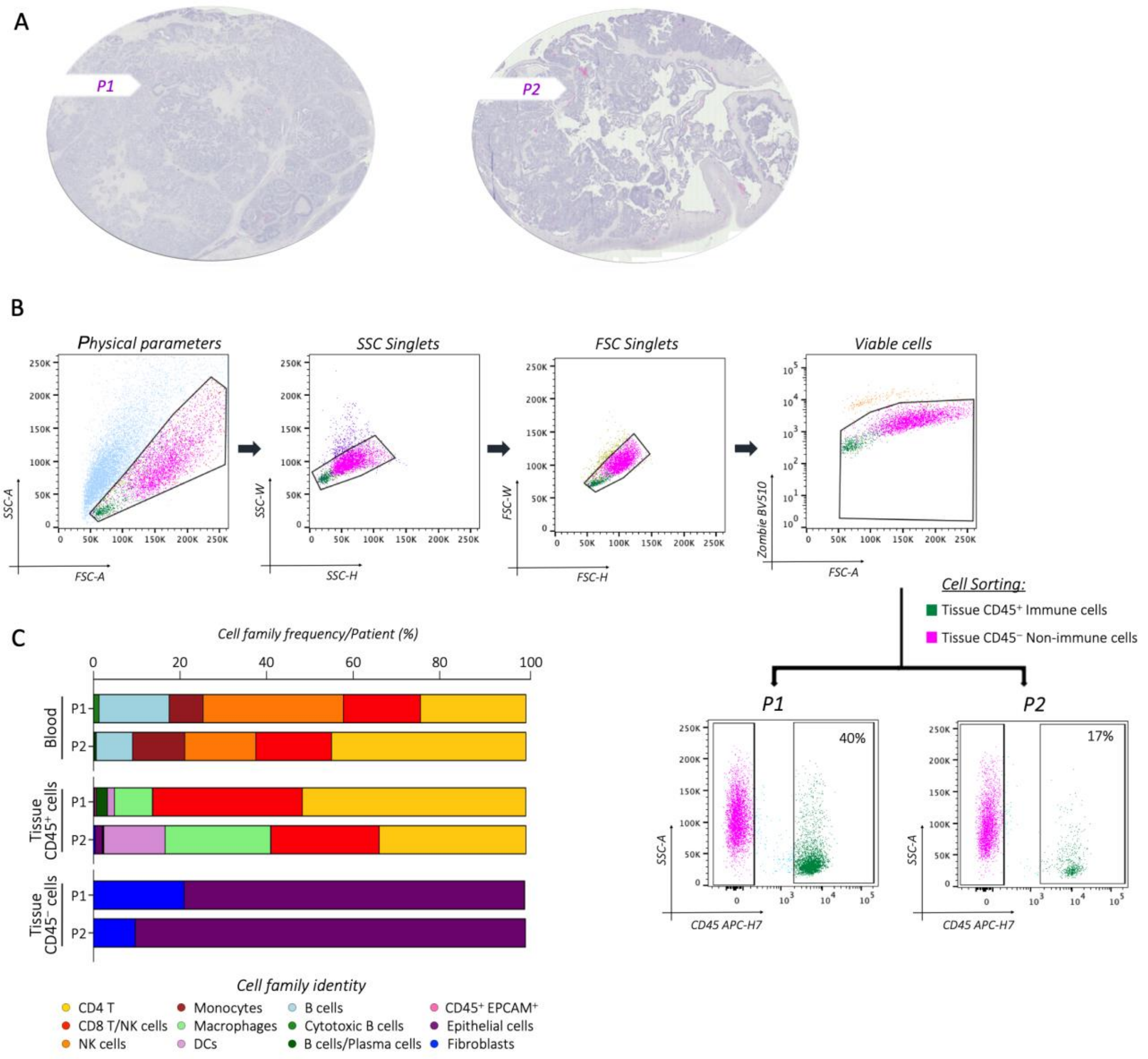

Figure S1. Comparative analysis of the stage I HGSOC microenvironment in two patients. **(A)** Representative HGSOC area showing hematoxylin and eosin (H&E) staining in patients P1 and P2. **(B)** Flow cytometry gating strategy employed to sort viable $CD45^+$ or $CD45^–$, with corresponding frequencies (%) in two patients P1 and P2. **(C)** Bar plot illustrating the relative frequencies (%) of different cell type distributions in blood- and tumor-derived $CD45^+$ and $CD45^–$ cells for patient P1 and P2. Cell numbers were normalized to the total loaded cell number for each patient and each tissue origin.

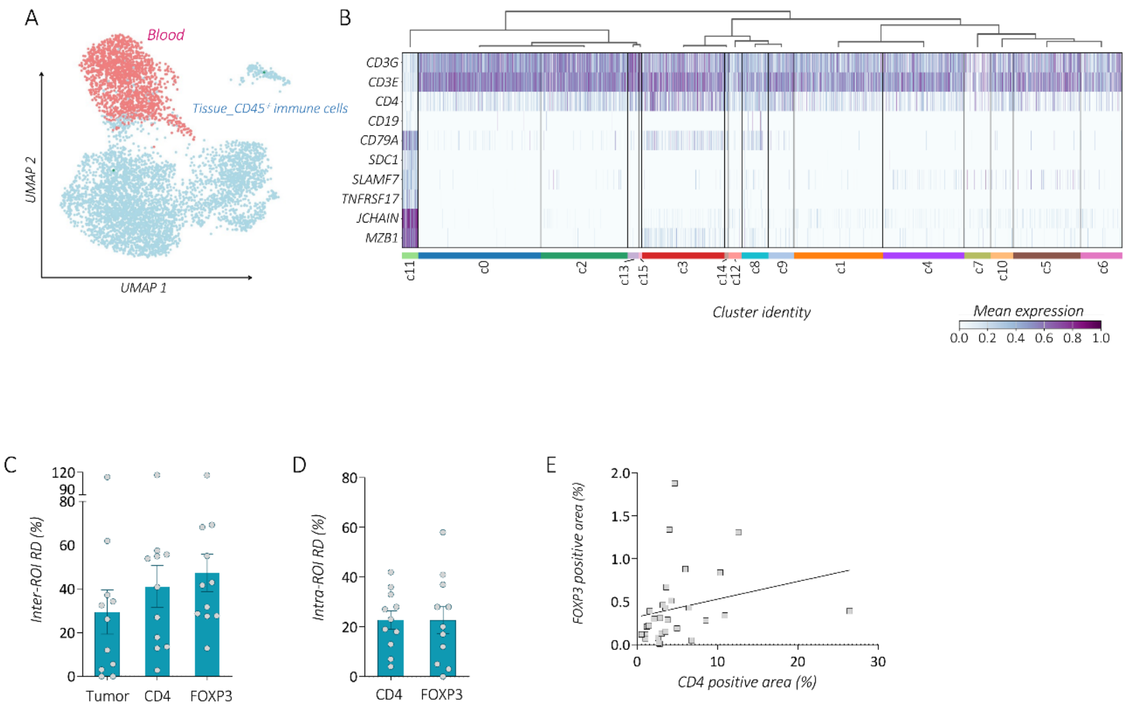

Figure S2. Detection of tumor-infiltrating CD4 T lymphocytes in stage I HGSOC.

(A) *UMAP* visualization of total re-clustered CD4 T cell subsets dissected by blood (red) and tumor (blue) origin. **(B)** Heatmap displaying the expression of key selected gene markers used for the exclusion of $CD3^{-}CD4^{-}$ contaminating cells in c11, detected among re-clustered CD4 T lymphocytes. Cells in c11 show Plasma cell profile, as indicated by the expression of *CD79A*, *JCHAIN*, and *SDC1*, along with low expression of *CD19*. **(C-D)** IHC study showing statistical analysis of the mean (±SEM) percentage (%) of the inter-ROI (*n=3* for each sample) **(C)** and the intra-ROI **(D)** relative dispersion (RD) detected in all samples (*n=11*). **(E)** Pearson correlation between matched $CD4^{+}$ and $FOXP3^{+}$ immunoreactive areas detected for each single ROI in all analyzed HGSOC samples (*n=11*).

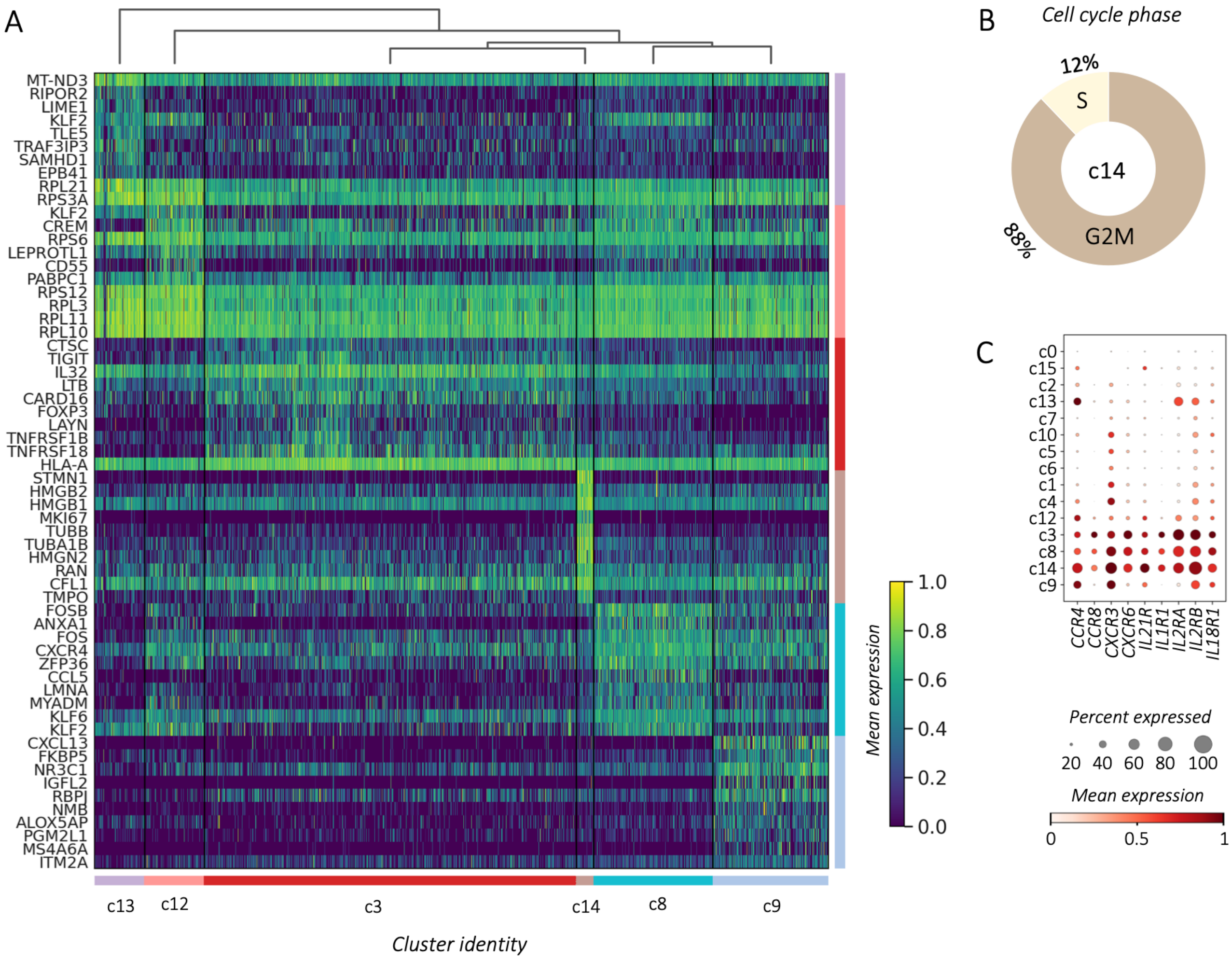

Figure S3. Transcriptomic diversity of Treg cell subsets in stage I HGSOC.
(A) Heatmap showing the top 10 DEGs for each blood and tumor-associated Treg subtype (c3-14/8-9/12-13). DEGs were defined as follows: (i) log2 FC ≥ 0.25; (ii) adjusted *P*-value (adj. p) < 0.05, and (iii) detected in at least 10% of cells (min. pct ≥ 10%). **(B)** Pie chart illustrating the relative frequency (%) of cell cycle phase G2M and S distribution among tumor-associated proliferating Tregs in c14. **(C)** Dot plot displaying the expression of cytokine receptors among all CD4 T cell clusters (c0-c15; *UMAP* Fig. 2A), selected based on detectable gene expression in tumor-associated Tregs. Dots are colored by the average expression of each gene scaled across all clusters and sized by the percentage of cells within a cluster (min.pct ≥ 10%).

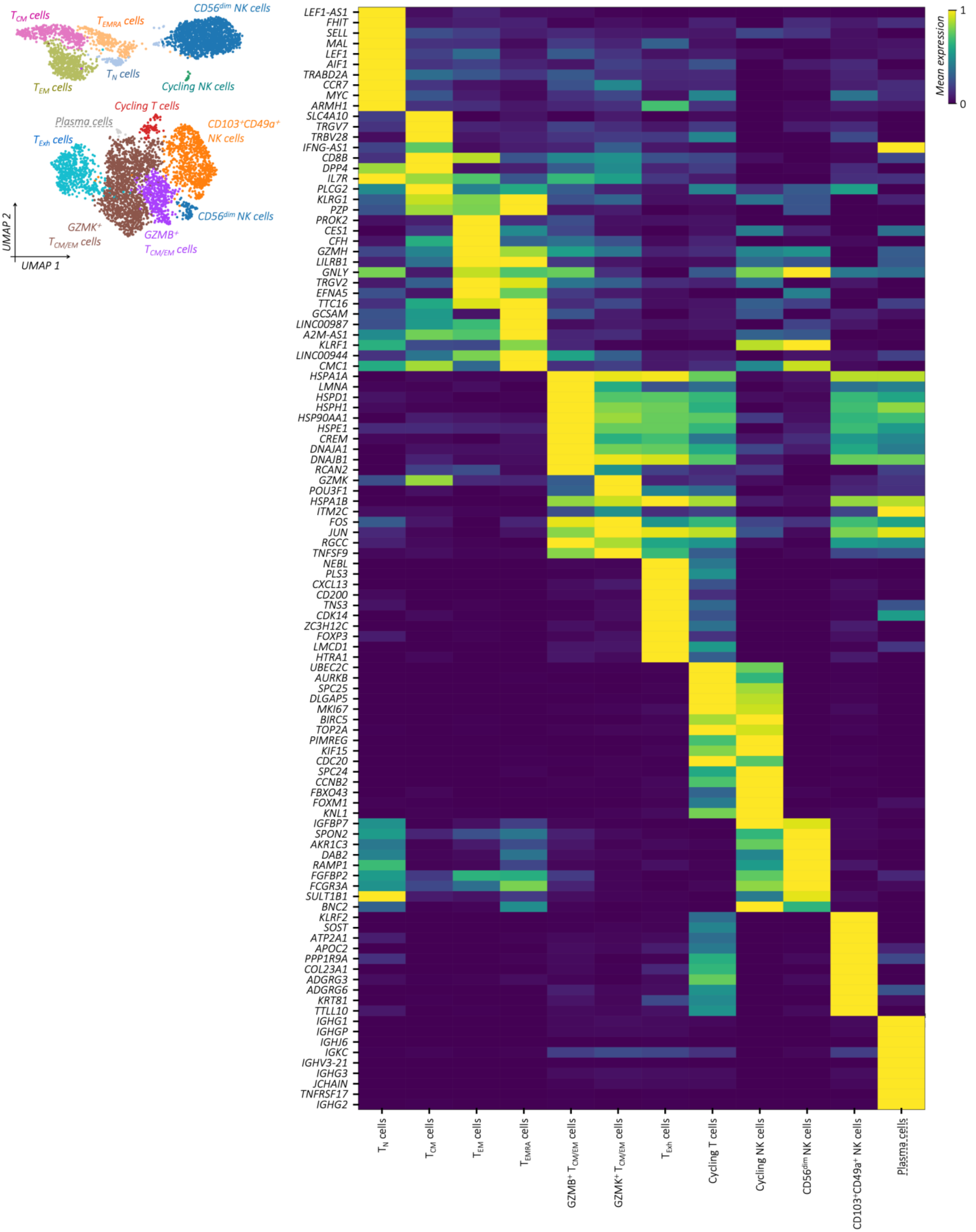
T_{CM} cells
T_{EMRA} cells
$CD56^{dim}$ NK cells
T_{EM} cells
T_N cells
Cycling NK cells
Cycling T cells
Plasma cells
$CD103^+CD49a^+$ NK cells
T_{Exh} cells
$CD56^{dim}$ NK cells
$GZMK^+$ $T_{CM/EM}$ cells
$GZMB^+$ $T_{CM/EM}$ cells
UMAP 2
UMAP 1
LEF1-AS1
FHIT
SELL
MAL
LEF1
AIF1
TRABD2A
CCR7
MYC
ARMH1
SLC4A10
TRGV7
TRBV28
IFNG-AS1
CD8B
DPP4
IL7R
PLCG2
KLRG1
PZP
PROK2
CES1
CFH
GZMH
LILRB1
GNLY
TRGV2
EFNA5
TTC16
GCSAM
LINC00987
A2M-AS1
KLRF1
LINC00944
CMC1
HSPA1A
LMNA
HSPD1
HSPH1
HSP90AA1
HSPE1
CREM
DNAJA1
DNAJB1
RCAN2
GZMK
POU3F1
HSPA1B
ITM2C
FOS
JUN
RGCC
TNFSF9
NEBL
PLS3
CXCL13
CD200
TNS3
CDK14
ZC3H12C
FOXP3
LMCD1
HTRA1
UBEC2C
AURKB
SPC25
DLGAP5
MKI67
BIRC5
TOP2A
PIMREG
KIF15
CDC20
SPC24
CCNB2
FBXO43
FOXM1
KNL1
IGFBP7
SPON2
AKR1C3
DAB2
RAMP1
FGFBP2
FCGR3A
SULT1B1
BNC2
KLRF2
SOST
ATP2A1
APOC2
PPP1R9A
COL23A1
ADGRG3
ADGRG6
KRT81
TTLL10
IGHG1
IGHGP
IGHJ6
IGKC
IGHV3-21
IGHG3
JCHAIN
TNFRSF17
IGHG2
T_N cells
T_{CM} cells
T_{EM} cells
T_{EMRA} cells
$GZMB^+$ $T_{CM/EM}$ cells
$GZMK^+$ $T_{CM/EM}$ cells
T_{Exh} cells
Cycling T cells
Cycling NK cells
$CD56^{dim}$ NK cells
$CD103^+CD49a^+$ NK cells
Plasma cells
Mean expression
1
0

Figure S4. Detection of cytotoxic lymphocytes in stage I HGSOC.
UMAP visualization of total re-clustered CD8 T and NK cell subsets dissected in blood and in the tumor (left panel). Heatmap (right panel) displaying the expression of the top 10 DEGs for each detected cell group, used for the exclusion of contaminating plasma cells (underlined dashed line) from further analysis of cytotoxic lymphocytes shown in Fig. 4. DEGs were defined as follows: (i) log2 FC ≥ 0.25; (ii) adjusted *P*-value (adj. p) ≤ 0.05, and (iii) detected in at least 10% of cells (min. pct ≥ 10%).

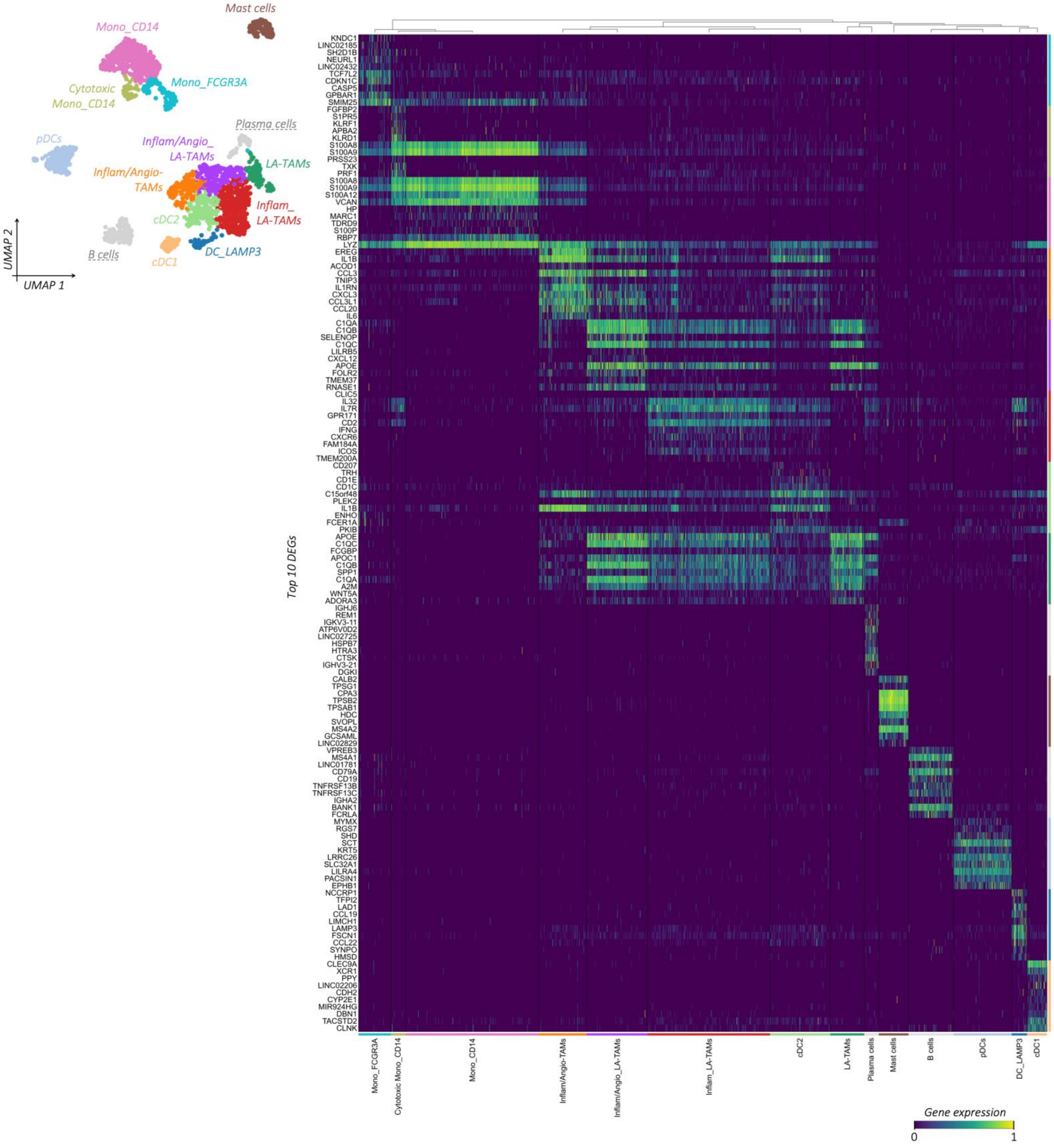
Mast cells
Mono_CD14
Mono_FCGR3A
Cytotoxic Mono_CD14
Plasma cells
pDCs
Inflam/Angio_ LA-TAMs
LA-TAMs
Inflam/Angio-TAMs
Inflam_ LA-TAMs
cDC2
B cells
cDC1
DC_LAMP3
UMAP 2
UMAP 1
Top 10 DEGs
KNDC1
LINC02185
SH2D1B
NEURL1
LINC02432
TCF7L2
CDKN1C
CASP5
GPBAR1
SMIM25
FGFBP2
S1PR5
KLRF1
APBA2
KLRD1
S100A8
S100A9
PRSS23
TXK
PRF1
S100A8
S100A9
S100A12
VCAN
HP
MARC1
TDRD9
S100P
RBP7
LYZ
EREG
IL1B
ACOD1
CCL3
TNIP3
IL1RN
CXCL3
CCL3L1
CCL20
IL6
C1QA
C1QB
SELENOP
C1QC
LILRB5
CXCL12
APOE
FOLR2
TMEM37
RNASE1
CLIC5
IL32
IL7R
GPR171
CD2
IFNG
CXCR6
FAM184A
ICOS
TMEM200A
CD207
TRH
CD1E
CD1C
C15orf48
PLEK2
IL1B
ENHO
FCER1A
PKIB
APOE
C1QC
FCGBP
APOC1
C1QB
SPP1
C1QA
A2M
WNT5A
ADORA3
IGHJ6
REM1
IGKV3-11
ATP6V0D2
LINC02725
HSPB7
HTRA3
CTSK
IGHV3-21
DGKI
CALB2
TPSG1
CPA3
TPSB2
TPSAB1
HDC
SVOPL
MS4A2
GCSAML
LINC02829
VPREB3
MS4A1
LINC01781
CD79A
CD19
TNFRSF13B
TNFRSF13C
IGHA2
BANK1
FCRLA
MYMX
RGS7
SHD
SCT
KRT5
LRRC26
SLC32A1
LILRA4
PACSIN1
EPHB1
NCCRP1
TFPI2
LAD1
CCL19
LIMCH1
LAMP3
FSCN1
CCL22
SYNPO
HMSD
CLEC9A
XCR1
PPY
LINC02206
CDH2
CYP2E1
MIR924HG
DBN1
TACSTD2
CLNK
Mono_FCGR3A
Cytotoxic Mono_CD14
Mono_CD14
Inflam/Angio-TAMs
Inflam/Angio_LA-TAMs
Inflam_LA-TAMs
cDC2
LA-TAMs
Plasma cells
Mast cells
B cells
pDCs
DC_LAMP3
cDC1
Gene expression
0
1

Figure S5. Detection of myeloid cells in stage I HGSOC.

UMAP visualization of total re-clustered myeloid cell subsets dissected in blood and in the tumor (left panel). Heatmap (right panel) displaying the expression of the top 10 DEGs for each detected cell group, used for the exclusion of contaminating plasma cells (underlined dashed line) from further analysis of myeloid cells shown in Fig. 5. DEGs were defined as follows: (i) log2 FC ≥ 0.25; (ii) adjusted *P*-value (adj. p) ≤ 0.05, and (iii) detected in at least 10% of cells (min. pct ≥ 10%).

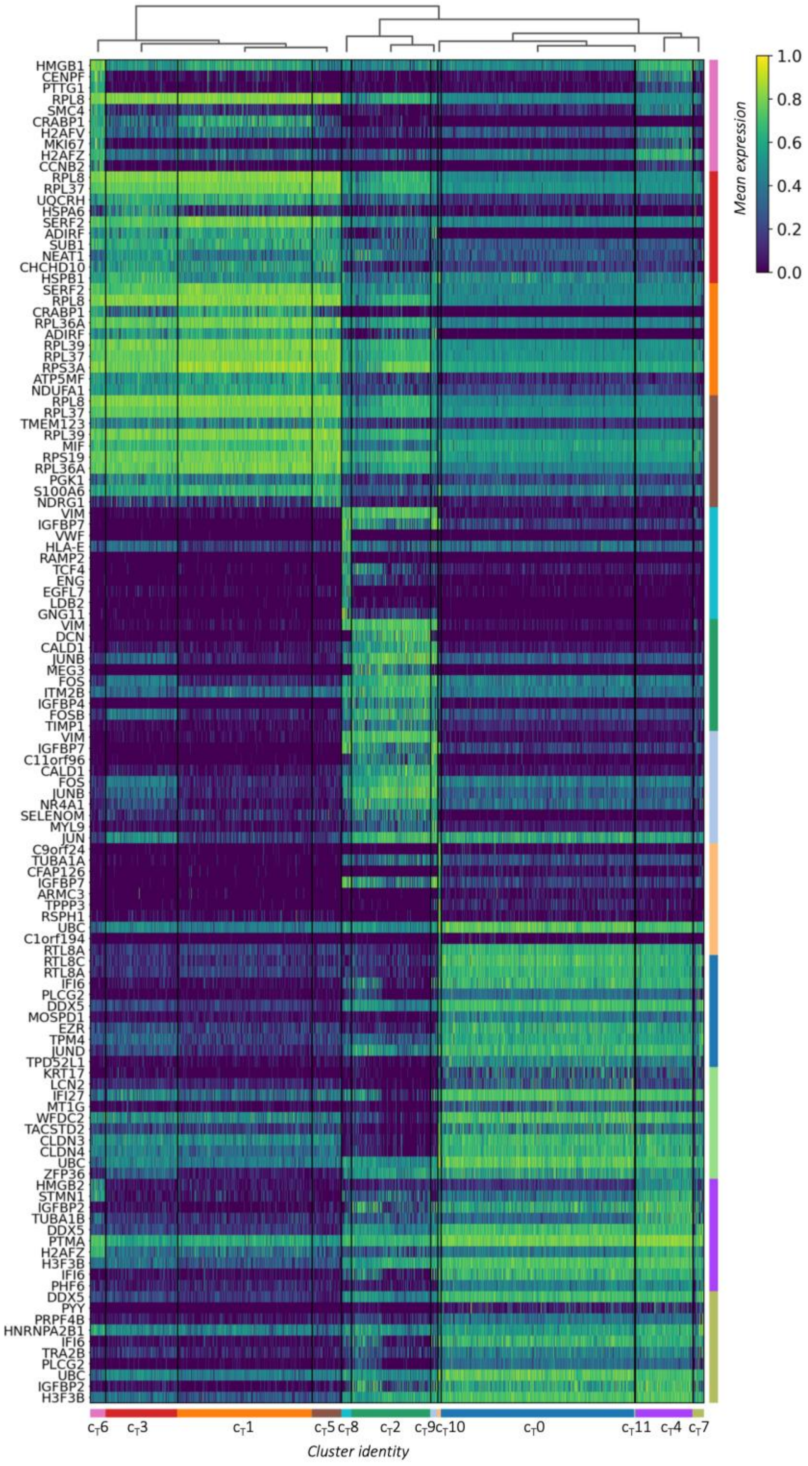

Figure S6. Transcriptomic patient-dependent diversity of tumor cells in stage I HGSOC. Heatmap showing the top 10 DEGs for each tumor-associated CD45⁻ cell cluster (C_T0-C_T11). DEGs were defined as follows: (i) log2 FC ≥ 0.25; (ii) adjusted *P*-value (adj. *P* < 0.05, and (iii) detected in at least 10% of cells (min. pct ≥ 10%).

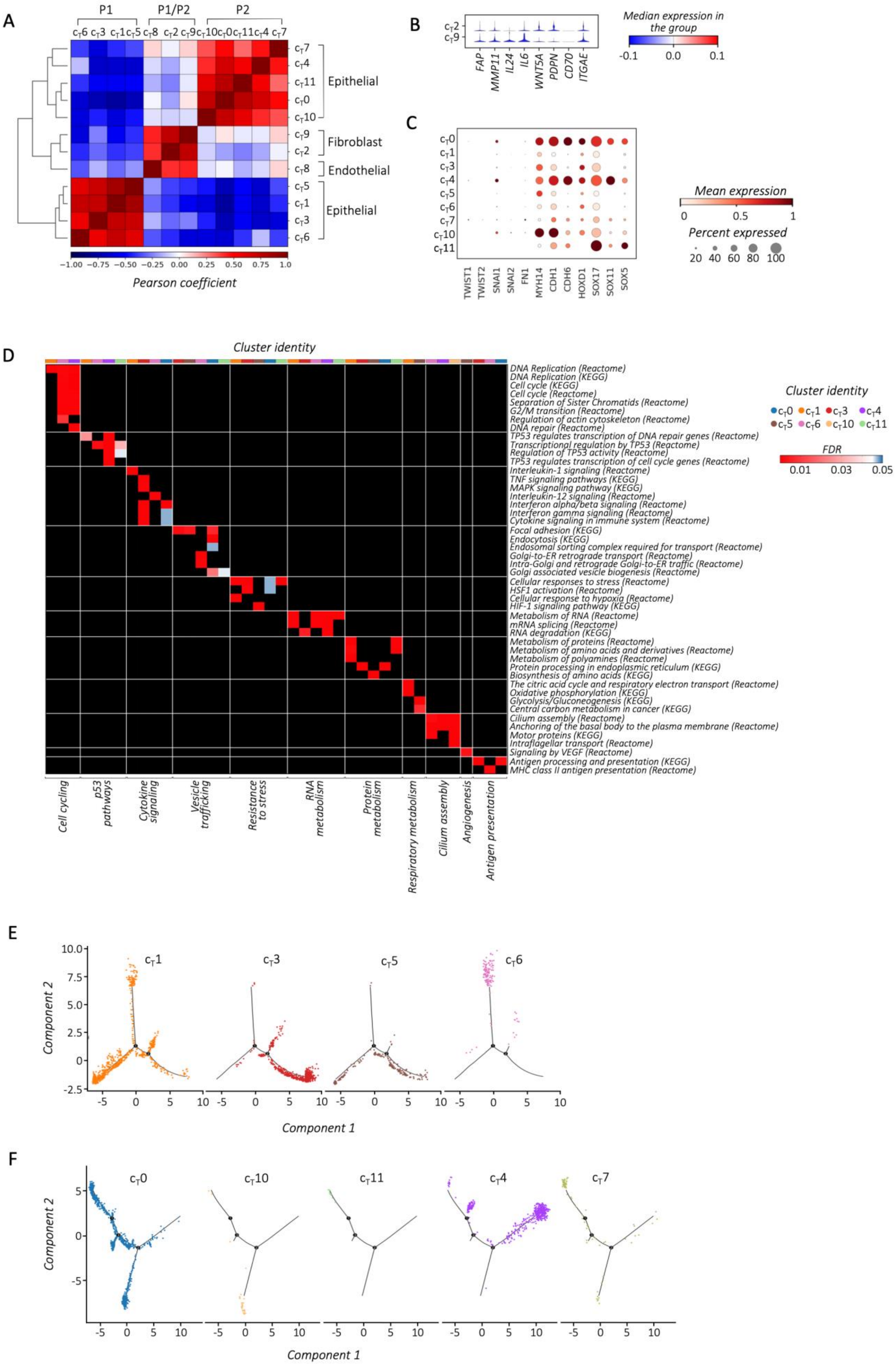
A
P1
P1/P2
P2
c_T6 c_T3 c_T1 c_T5 c_T8 c_T2 c_T9 c_T10 c_T0 c_T11 c_T4 c_T7
Epithelial
Fibroblast
Endothelial
Epithelial
−1.00 −0.75 −0.50 −0.25 0.00 0.25 0.50 0.75 1.0
Pearson coefficient
B
c_T2
c_T9
FAP MMP11 IL24 IL6 WNT5A PDPN CD70 ITGAE
Median expression in the group
-0.1 0.0 0.1
C
c_T0 c_T1 c_T3 c_T4 c_T5 c_T6 c_T7 c_T10 c_T11
TWIST1 TWIST2 SNAI1 SNAI2 FN1 MYH14 CDH1 CDH6 HOXD1 SOX17 SOX11 SOX5
Mean expression
0 0.5 1
Percent expressed
20 40 60 80 100
D
Cluster identity
DNA Replication (Reactome)
DNA Replication (KEGG)
Cell cycle (KEGG)
Cell cycle (Reactome)
Separation of Sister Chromatids (Reactome)
G2/M transition (Reactome)
Regulation of actin cytoskeleton (Reactome)
DNA repair (Reactome)
TP53 regulates transcription of DNA repair genes (Reactome)
Transcriptional regulation by TP53 (Reactome)
Regulation of TP53 activity (Reactome)
TP53 regulates transcription of cell cycle genes (Reactome)
Interleukin-1 signaling (Reactome)
TNF signaling pathways (KEGG)
MAPK signaling pathway (KEGG)
Interleukin-12 signaling (Reactome)
Interferon alpha/beta signaling (Reactome)
Interferon gamma signaling (Reactome)
Cytokine signaling in immune system (Reactome)
Focal adhesion (KEGG)
Endocytosis (KEGG)
Endosomal sorting complex required for transport (Reactome)
Golgi-to-ER retrograde transport (Reactome)
Intra-Golgi and retrograde Golgi-to-ER traffic (Reactome)
Golgi associated vesicle biogenesis (Reactome)
Cellular responses to stress (Reactome)
HSF1 activation (Reactome)
Cellular response to hypoxia (Reactome)
HIF-1 signaling pathway (KEGG)
Metabolism of RNA (Reactome)
mRNA splicing (Reactome)
RNA degradation (KEGG)
Metabolism of proteins (Reactome)
Metabolism of amino acids and derivatives (Reactome)
Metabolism of polyamines (Reactome)
Protein processing in endoplasmic reticulum (KEGG)
Biosynthesis of amino acids (KEGG)
The citric acid cycle and respiratory electron transport (Reactome)
Oxidative phosphorylation (KEGG)
Glycolysis/Gluconeogenesis (KEGG)
Central carbon metabolism in cancer (KEGG)
Cilium assembly (Reactome)
Anchoring of the basal body to the plasma membrane (Reactome)
Motor proteins (KEGG)
Intraflagellar transport (Reactome)
Signaling by VEGF (Reactome)
Antigen processing and presentation (KEGG)
MHC class II antigen presentation (Reactome)
Cluster identity
c_T0 c_T1 c_T3 c_T4
c_T5 c_T6 c_T10 c_T11
FDR
0.01 0.03 0.05
Cell cycling
p53 pathways
Cytokine signaling
Vesicle trafficking
Resistance to stress
RNA metabolism
Protein metabolism
Respiratory metabolism
Cilium assembly
Angiogenesis
Antigen presentation
E
c_T1 c_T3 c_T5 c_T6
10.0 7.5 5.0 2.5 0 -2.5
Component 2
-5 0 5 10
Component 1
F
c_T0 c_T10 c_T11 c_T4 c_T7
5 0 -5
Component 2
-5 0 5 10
Component 1

Figure S7. Transcriptomic profiling of tumor cells in stage I HGSOC.

(A) Heatmap displaying the *Pearson* correlation matrix for different tumor-associated CD45^{-} cell clusters (C_T0-C_T11). **(B)** Violin plot showing the expression of key selected gene markers depicting the fibroblast malignant transformation in C_T2 and C_T9. **(C)** Dot plot showing the expression of selected gene markers depicting the mesenchymal transformation profile among malignant epithelial cell clusters C_T0-1/3-7/10-11. Dots are colored by the average expression of each gene scaled across all clusters and sized by the percentage of cells within a cluster (min.pct ≥ 10%). **(D)** Heatmap displaying the selection of significantly enriched *Reactome* and *KEGG* pathways with *FDR*-value < 0.05 (*Reactome*) or *q*-value (*KEGG*) < 0.05, identified among DEGs. **(E-F)** Pseudotime trajectories projecting single tumor cell clusters (C_T0-1/3-7/10-11) (left panels), for patient P1 **(E)** and P2 **(F)**.

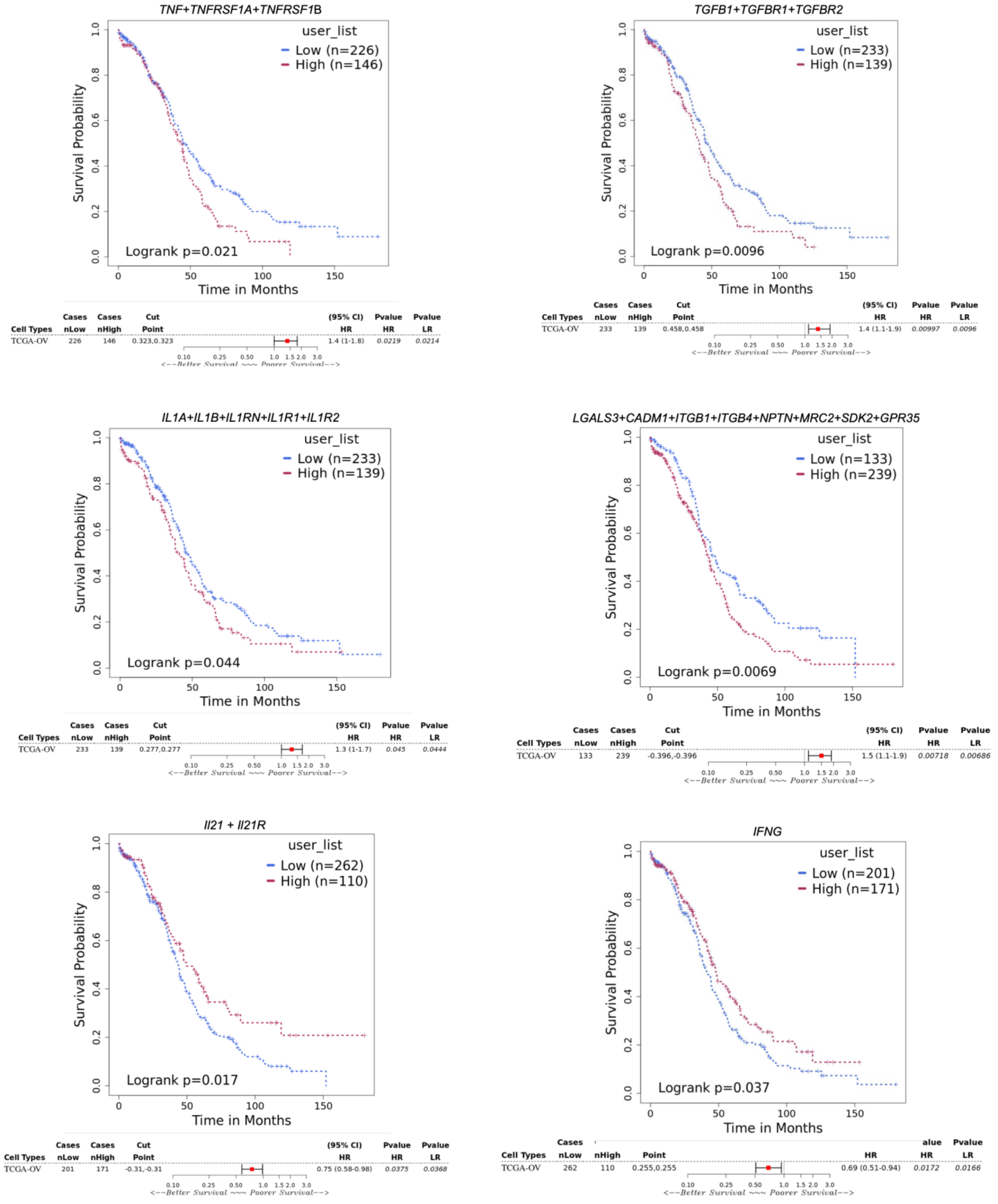
TNF+TNFRSF1A+TNFRSF1B
user_list
Low (n=226)
High (n=146)
Logrank p=0.021
Survival Probability
Time in Months
Cell Types | Cases nLow | Cases nHigh | Cut Point | (95% CI) HR | Pvalue HR | Pvalue LR
TCGA-OV | 226 | 146 | 0.323,0.323 | 1.4 (1-1.8) | 0.0219 | 0.0214
TGFB1+TGFBR1+TGFBR2
user_list
Low (n=233)
High (n=139)
Logrank p=0.0096
Survival Probability
Time in Months
Cell Types | Cases nLow | Cases nHigh | Cut Point | (95% CI) HR | Pvalue HR | Pvalue LR
TCGA-OV | 233 | 139 | 0.458,0.458 | 1.4 (1.1-1.9) | 0.00997 | 0.0096
IL1A+IL1B+IL1RN+IL1R1+IL1R2
user_list
Low (n=233)
High (n=139)
Logrank p=0.044
Survival Probability
Time in Months
Cell Types | Cases nLow | Cases nHigh | Cut Point | (95% CI) HR | Pvalue HR | Pvalue LR
TCGA-OV | 233 | 139 | 0.277,0.277 | 1.3 (1-1.7) | 0.045 | 0.0444
LGALS3+CADM1+ITGB1+ITGB4+NPTN+MRC2+SDK2+GPR35
user_list
Low (n=133)
High (n=239)
Logrank p=0.0069
Survival Probability
Time in Months
Cell Types | Cases nLow | Cases nHigh | Cut Point | (95% CI) HR | Pvalue HR | Pvalue LR
TCGA-OV | 133 | 239 | -0.396,-0.396 | 1.5 (1.1-1.9) | 0.00718 | 0.00686
Il21 + Il21R
user_list
Low (n=262)
High (n=110)
Logrank p=0.017
Survival Probability
Time in Months
Cell Types | Cases nLow | Cases nHigh | Cut Point | (95% CI) HR | Pvalue HR | Pvalue LR
TCGA-OV | 201 | 171 | -0.31,-0.31 | 0.75 (0.58-0.98) | 0.0375 | 0.0368
IFNG
user_list
Low (n=201)
High (n=171)
Logrank p=0.037
Survival Probability
Time in Months
Cell Types | Cases nLow | nHigh | Point | HR | alue HR | Pvalue LR
TCGA-OV | 262 | 110 | 0.255,0.255 | 0.69 (0.51-0.94) | 0.0172 | 0.0166
<--Better Survival ~~~ Poorer Survival-->

Figure S8. Clinical relevance of the predicted Treg cell-interaction network in stage I HGSOC.

Kaplan-Meier curves with corresponding Forest plots for patients with advanced OC (n=372; TCGA dataset), showing significantly worse OS prognosis between high-risk and low-risk expression levels of specific receptor-ligand interaction axes detected in $FOXP3^{high}$ and $FOXP3^{+}$ Tregs: *TNF-TNFRSF1A/1B*, *TGFB1-TGFBR1/R2*, *IL1A/1B-IL1RN/IL1R1/R2*, and *LGALS3-CADM1/ITGB1/ITGB4/NPTN/MRC2/SDK2/GPR35*, *IL21-IL21R* and expression of *IFNG*. Significant differences between groups are indicated by the *P*-value: **P*-value < 0.05.

Supplementary Tables

Table S1. Cell cycle phase gene list.

Cell cycle phase genes			
MCM5	*TOP2A*	*WDR76*	*HJURP*
PCNA	*NDC80*	*SLBP*	*CDCA3*
TYMS	*CKS2*	*CCNE2*	*HN1*
FEN1	*NUF2*	*UBR7*	*CDC20*
MCM2	*CKS1B*	*POLD3*	*TTK*
MCM4	*MKI67*	*MSH2*	*CDC25C*
RRM1	*TMPO*	*ATAD2*	*KIF2C*
UNG	*CENPF*	*RAD51*	*RANGAP1*
GINS2	*TACC3*	*RRM2*	*NCAPD2*
MCM6	*FAM64A*	*CDC45*	*DLGAP5*
CDCA7	*SMC4*	*CDC6*	*CDCA2*
DTL	*CCNB2*	*EXO1*	*CDCA8*
PRIM1	*CKAP2L*	*TIPIN*	*ECT2*
UHRF1	*CKAP2*	*DSCC1*	*KIF23*
MLF1IP	*AURKB*	*BLM*	*HMMR*
HELLS	*BUB1*	*CASP8AP2*	*AURKA*
RFC2	*KIF11*	*USP1*	*PSRC1*
RPA2	*ANP32E*	*CLSPN*	*ANLN*
NASP	*TUBB4B*	*POLA1*	*LBR*
RAD51AP1	*GTSE1*	*CHAF1B*	*CKAP5*
GMNN	*KIF20B*	*BRIP1*	*CENPE*
WDR76	*HJURP*	*E2F8*	*CTCF*
SLBP	*CDCA3*	*HMGB2*	*NEK2*
CCNE2	*HN1*	*CDK1*	*G2E3*
UBR7	*CDC20*	*NUSAP1*	*GAS2L3*
POLD3	*TTK*	*UBE2C*	*CBX5*
MSH2	*CDC25C*	*BIRC5*	*CENPA*
ATAD2	*KIF2C*	*TPX2*	

Table S2. Gene-score signatures used to compute the Lipid-Associated (LA), Angiogenesis (Angio) and Inflammatory (Inflam) effector scores in myeloid cells.

Lipid Associated (LA) score	Angiogenesis (Angio) score	Inflammatory (Inflam) score
ACP5	*CCL2*	*CCL2*
APOE	*CCL4*	*CCL3*
APOC1	*CCL20*	*CCL4*
C1QA	*CD163*	*CCL5*
C1QB	*CD300E*	*CCL20*
C1QC	*CD44*	*CCL3L1*
TREM2	*CD55*	*CCL4L2*
CTSL	*CLEC5A*	*CXCL1*
F13A1	*EREG*	*CXCL2*
FOLR2	*FLT1*	*CXCL3*
GPNMB	*FN1*	*CXCL8*
LIPA	*HES1*	*G0S2*
MMP9	*IL1B*	*IL1B*
MRC1	*IL1RN*	*IL1RN*
NR1H3	*CXCL8*	*IL6*
NUPR1	*OLR1*	*INHBA*
PLA2G7	*THBS1*	*PMAIP1*
RNASE1	*VEGFA*	*IL32*
MAF	*BACH1*	*IL18*
	CSF1R	*CXCL12*
		CXCL16

Table S3. Specific tumor cell cluster-associated signature in stage I HGSOC patients P1 and P2. Cluster specific gene sets were obtained through DE analysis between all epithelial cell clusters. The top 35 DEGs for each cluster were selected based on adjusted *P* value < 0.01 and Log2 Fold-Change > 0.25.

P1 Cluster identity	P2 Cluster identity
**c_T5**	**c_T0**
NDUFA4L2	*MALL*
IGFBP3	*TGFA*
EGLN3	*GPRC5A*
CA12	*RNF223*
KISS1R	*ZBED2*
CAV1	*ERP27*
KDR	*TLR5*
PNCK	*TGM2*
NDRG1	*TPBG*
CAV2	*TMEM150A*
ENO2	*ALPP*
SLC6A8	*SCGB2A1*
ANGPTL4	*MMEL1*
AHNAK2	*MMP7*
S100A10	*ZNF750*
NXPH4	*TNFRSF14*
HILPDA	*KDM7A*
P4HA1	*ITIH5*
QSOX1	*AC007009.1*
VEGFA	*DHRS3*
CASC8	*B4GALT5*
SPAG4	*PRUNE2*
GCLC	*FXYD5*
FRY	*AHNAK2*
SLC2A1	*RAB25*
S100A4	*ALDH3B1*
MACC1	*LDLRAD1*
BNIP3L	*MUC20*
PLIN2	*MLPH*
ARHGDIB	*EMP1*
LTBP1	*NRP2*
CH25H	*HLA-DRB1*
CDKN2B	*GRAMD1C*
PFKP	*PPP1R3B*
FAM13A	*CLIC3*
